# Using Paleogenomics to Study the Evolution of Gene Families: Origin and Duplication History of the Relaxin Family Hormones and Their Receptors

**DOI:** 10.1371/journal.pone.0032923

**Published:** 2012-03-21

**Authors:** Sergey Yegorov, Sara Good

**Affiliations:** Department of Biology, University of Winnipeg, Winnipeg, Manitoba, Canada; Hebrew University at Jerusalem, - The Alexander Silberman Institute of Life Sciences, Israel

## Abstract

Recent progress in the analysis of whole genome sequencing data has resulted in the emergence of paleogenomics, a field devoted to the reconstruction of ancestral genomes. Ancestral karyotype reconstructions have been used primarily to illustrate the dynamic nature of genome evolution. In this paper, we demonstrate how they can also be used to study individual gene families by examining the evolutionary history of relaxin hormones (RLN/INSL) and relaxin family peptide receptors (RXFP). Relaxin family hormones are members of the insulin superfamily, and are implicated in the regulation of a variety of primarily reproductive and neuroendocrine processes. Their receptors are G-protein coupled receptors (GPCR's) and include members of two distinct evolutionary groups, an unusual characteristic. Although several studies have tried to elucidate the origins of the relaxin peptide family, the evolutionary origin of their receptors and the mechanisms driving the diversification of the RLN/INSL-RXFP signaling systems in non-placental vertebrates has remained elusive. Here we show that the numerous vertebrate *RLN/INSL* and *RXFP* genes are products of an ancestral receptor-ligand system that originally consisted of three genes, two of which apparently trace their origins to invertebrates. Subsequently, diversification of the system was driven primarily by whole genome duplications (WGD, 2R and 3R) followed by almost complete retention of the ligand duplicates in most vertebrates but massive loss of receptor genes in tetrapods. Interestingly, the majority of 3R duplicates retained in teleosts are potentially involved in neuroendocrine regulation. Furthermore, we infer that the ancestral AncRxfp3/4 receptor may have been syntenically linked to the AncRln-like ligand in the pre-2R genome, and show that syntenic linkages among ligands and receptors have changed dynamically in different lineages. This study ultimately shows the broad utility, with some caveats, of incorporating paleogenomics data into understanding the evolution of gene families.

## Introduction

**Figure 1:**
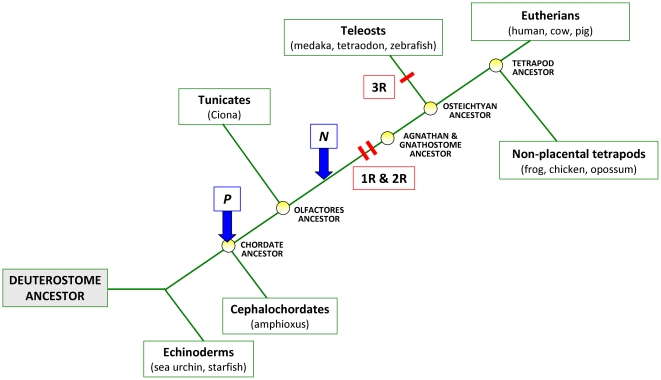
Simplified phylogenetic tree showing the evolutionary relationships among the major deuterostome taxa (with the common names of organisms in brackets) and their ancestors discussed in this paper. The hypothetical ancestral genome predicted by Nakatani *et al*. (“N”) probably belongs to an organism that existed just before 2R in early vertebrates. On the other hand, Putnam *et al*. (“P”) reconstructed the karyotype of the common ancestor of amphioxus and the olfactores (tunicates+vertebrates). 1R, 2R and 3R mark the three rounds of whole genome duplication that happened throughout vertebrate evolution. Tree topology adapted from Putnam *et al* [20].

Analyses of whole genome sequence data have confirmed that three rounds of whole genome duplication (WGD) are thought to have contributed immensely to the diversification of vertebrates [1], [2], [3]; two rounds of WGD (2R) occurred in early vertebrate evolution, probably before the divergence of agnathans and gnathostomes [4], while the third round (3R) occurred at the base of the teleostean lineage (Figure 1). Even though gene duplication has long been recognized as a major factor in the evolution of biological diversity [5], [6], determining the evolutionary relationships among members of gene families is not always easy because individual genes originate via both small-scale and whole genome duplication events, can be modified by selection or concerted evolution, and may experience differential loss across lineages [5], [6], [7], [8]. Although the ready availability of small-scale synteny data has facilitated the determination of orthologous and paralogous relationships among genes, some aspects of gene family evolution, such as their ancient origins and the timing and kind of duplication events they underwent, continue to be difficult to resolve using traditional bioinformatic approaches [9], [10].

Recently, large scale synteny analyses comparing entire genomes of evolutionarily distant taxa have been employed to reconstruct the karyotypes of extinct ancestors and to look back at the events that shaped the appearance of modern genomes [11]. Ancestral genome reconstruction models depict metazoan chromosomes as composed of segments, originating from one or more linkage groups of a distant ancestor, which become united following repeated chromosomal fission and fusion events to ultimately form the karyotypes of modern taxa. By tracing the syntenic relationships among such chromosomal segments from two or more extant taxa, it is possible to reconstruct the linkage groups of their common ancestor at the time of taxon divergence. For example, comparison of the genomes of tetrapods and teleosts allows one to infer the chromosomes of the hypothetical ∼450 MY old gnathostome ancestor and to outline the linkage groups of the ∼500 MY old ancestor of all extant vertebrates (Figure 1) [12].

Reconstructions of ancestral genomes in the chordate lineage are particularly interesting, because they shed light on the role of WGD events and the intensive karyotype rearrangements that played key roles in the evolution of the vertebrate genetic portfolio. Although it has been suggested that genome reconstructions provide principally a heuristic tool for understanding genome evolution [11], in this study we show how such models can be used to trace the evolutionary history and linkage relationship of genes, thereby giving further power to elucidate both the origin and duplication history of gene families. To demonstrate the utility of this approach, we focus on the origins of a group of small peptide hormones and their receptors, whose evolutionary history has been a matter of debate.

The relaxin (RLN) and insulin-like (INSL) peptides mediate a broad variety of primarily reproductive and neuroendocrine functions and are hypothesized to have played important roles in mammalian evolution [13]. The relaxin-family hormones belong to the Insulin-Relaxin superfamily, which also includes Insulin (INS) and Insulin-like growth factors (IGF). However, distinct from both INS and IGF, which signal via receptor tyrosine kinases (RTK), RLN/INSL peptides interact with two very dissimilar classes of G protein-coupled receptors (GPCR). One class of Relaxin family peptide receptors (RXFPs), consisting of RXFP1 and RXFP2 (also known as LGR7 and LGR8 respectively), is closely related to glycoprotein hormone receptors and is hence distinguished by a large N-terminus containing leucine-rich repeats [14]. The other class of RXFPs, consisting of RXFP3 and RXFP4 (also known as SALPR and GPCR142), is related to small peptide (e.g. angiotensin or somatostatin) receptors, which have relatively short N-termini [14]. The promiscuous interaction of RLN/INSL with these 2 diverse classes of receptors is rare among GPCR ligands and its evolutionary significance has yet to be clarified [15].

Although it has been shown that orthologous copies of four *RLN/INSL* genes (*RLN, INSL3, INSL5* and *RLN3*) are present in teleosts and mammals, the exact mechanisms giving rise to their diversification in non-placental vertebrates have remained elusive [13], [16], [17], [18]. For instance, it was proposed that the signaling system originated via duplication of the *INS/IGF* locus in the ancestor of olfactores (tunicates+vertebrates), and that the ancestral ligand originally functioned using an insulin RTK-like receptor, prior to switching to an RXFP3/4-type receptor in early vertebrates [19]. At the same time it was believed that RXFP1/2 receptors were not recruited into the system until after the emergence of mammals [13]. In addition, it has also been hypothesized that 3 vertebrate *RLN/INSL* genes are ohnologs, i.e. products of 2R that took place in the vertebrate ancestor, while the fourth gene arose from a local duplication [17].

In this paper, we employ paleogenomics models to look at the origin and linkage relationships of *RLN/INSL* and *RXFP* genes and to determine the role of WGDs in their diversification. We provide evidence that WGDs played a central role, larger than previously appreciated, in the origination of these gene families and suggest that the system consisted of a trio of 2 receptors (one RXFP1/2-like and the other RXFP3/4-like) and a single ligand in the vertebrate ancestor. We find support for the hypotheses generated from the ancestral genome reconstruction models by employing small-scale synteny analyses and phylogenetic reconstructions performed on a broad repertoire of focal genes, and ultimately show the utility, with some caveats, of incorporating paleogenomics data into understanding the evolution of gene families.

## Results

**Figure 2:**
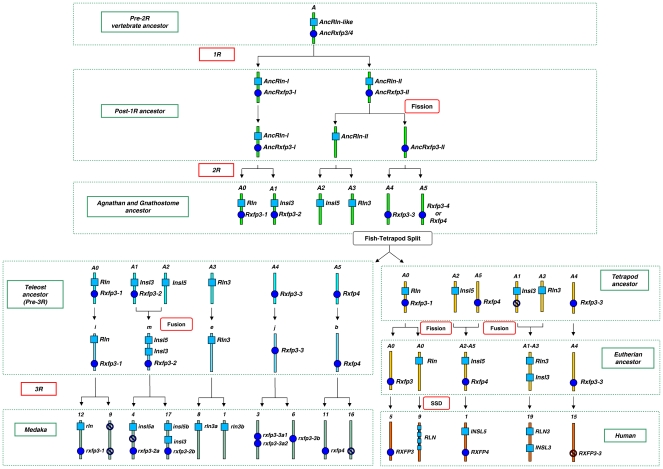
Reconstruction of the genetic events that led to the diversification of RXFP3-type receptors and RLN/INSL hormones in vertebrates based on the “fission” scenario of ancestral genome rearrangement. The genomic origins of the hypothetical ancestral relaxin (*AncRln-like*) and *Rxfp3/4* receptor (*AncRxfp3/4*) genes can be traced to a single chromosome in the vertebrate ancestor that had not yet been through 2R (Pre-2R vertebrate ancestor). The ancestral linkage group harboring *AncRln-like* and *AncRxfp3/4-like* sequentially underwent duplication, fission and another duplication yielding 5 distinct linkage groups (agnathan and gnathostome ancestor) harboring the ligand and receptor genes. Subsequently, tetrapods completely lost *RXFP3-2* and often *RXFP3-3* genes, but retained all of the post-2R ligand gene duplicates. Teleosts, on the other hand, retained both all of the ligand and receptor post-2R gene duplicates, suggesting that *RXFP3-2* and *RXFP3-3* acquired important functions in the pre-3R teleost ancestor. The duplicates of *rxfp3-2* and *rxfp3-3* were again retained in the post-3R teleost ancestor along with those of *rln3* and *insl5* (indicating their possible ligand-receptor relationships). Lastly, in vertebrates the *RLN* locus underwent multiple local duplications, resulting in the emergence of *INSL4* in all eutherians, and *INSL6* and *RLN1* only in apes, whose *RLN2* is orthologous to *RLN* of other eutherians. For simplicity, tetrapod and eutherian ancestor linkage groups are only shown to contain the fragments (e.g. *A0, A2–A5*) harboring the genes of interest; thus they should not be confused with actual chromosomes. Blue circles and squares represent receptor and their ligand genes respectively. Crossed circles represent pseudogenes (red, if they are verified in databases, blue if they are hypothetical). SSD: small-scale duplication. The first letter of ancestral gene names is capitalized.

In the first part of this study, we inferred the origins of the *RLN/INSL* and *RXFP* gene sets by comparing the ancestry of large chromosomal fragments in a teleost fish (Japanese medaka), a bird (chicken) and human using a model of vertebrate genome evolution [12], the “N-model” (for a full explanation of the method, see Supporting Information S1). Since, with some exceptions, *RLN/INSL* and *RXFP* genes in non-mammals have been primarily characterized by automated gene scan tools and are poorly annotated, we searched a number of available vertebrate genomes (25 species) for our focal genes (235 total genes) to ensure that we considered all potential ligand and receptor ohnologs (see Dataset S1: Tables S4–S8). Thus for human, chicken and medaka, we mapped the genomic positions of 4, 3 and 6 ligand *RLN/INSL* and 6, 4 and 9 receptor *RXFP* genes (or pseudogenes) respectively onto the linkage groups contained in each of the 3 vertebrate genomes (according to the N-model) and “traced” their origins to the gnathostome ancestor chromosomes (*GAC*), i.e. linkage groups of the hypothetical post-2R ancestor of jawed (and possibly jawless, see [4]) vertebrates. According to the N-model, each of the 40 post-2R reconstructed *GAC*s (*A0-J1*) originate from 10–13 Vertebrate Ancestral Chromosomes (*VAC, A-J*), i.e. linkage groups that existed in the hypothetical pre-2R genome. For 3 of the *VACs* (*A*, *B* and *F*), Nakatani *et al*. [12] were able to reconstruct the major chromosomal events that established chromosomal content of the post-1R and -2R vertebrate ancestor genomes. The occurrence of several of our genes-of-interest on these *GAC*s allowed us to not only trace their pre-2R origins, but also to assess the number and linkage relationships of the *RLN/INSL* and *RXFP* genes in the intermediate post-1R vertebrate ancestor. In their work, Nakatani *et al*. [12] proposed two alternative scenarios for the duplication and rearrangement history of *VAC* “*A*” (found to host the predecessors of both *RLN/INSL* and *RXFP3/4* genes, see below). We considered both scenarios (Figures 2 and S1) and adopted here the one presented in the main text of Nakatani *et al* [10], which also minimizes the number of linkage groups in the pre-2R vertebrate ancestor. As described in detail in Supporting Information S1, the primary difference of the main (“fusion”) and the alternative (“fission”) models concerns the syntenic linkage of *AncRln-like* and *AncRxfp3/4* genes.

### *RLN/INSL* and *RXFP3/4* originate from ancestral linkage group *VAC* “*A*”, while *RXFP1/2* originates from another group, *VAC* “*C*”

Our analyses revealed that *RLN*, *RLN3*, *INSL3*, *INSL5* and their orthologs in teleosts originated from one location in *VAC “A”* in the pre-2R vertebrate ancestor (Dataset S1: Table S1). Since each of the four *RLN/INSL* genes can be mapped to 4 distinct 2R-derived *GAC*s (*A0*, *A1*, *A2* and *A3*), we infer that modern vertebrate relaxin family genes arose from a single ancestral gene, *AncRln-like*, as a result of 2R (Figure 2).

The origins of the receptor *RXFP3/4* genes in tetrapods and teleosts were traced to four *GAC*s (*A0*, *A1*, *A4* and *A5*; 2 of which, *A0* and *A1*, are the same as those hosting *RLN* and *INSL3*), which suggests that they also originated from one gene, *AncRxfp3/4-like*, located on *VAC* “*A*” (Dataset S1: Table S1). This indicates that the ancestral genes for *RLN/INSL* and *RXFP3/4* were physically linked before 2R took place (Figure 2; whereas in the alternative scenario, the ancestral ligand and receptor genes were located in separate linkage groups and half of their duplicates became linked in the post-1R descendants, Figure S2 and Supporting Information S1).

The high number of receptor *rxfp3*-type genes in teleosts is explained by the post-2R retention of all four *rxfp3/4* ohnologs in the teleost ancestor. Additionally, the fish-specific 3R coupled with a few local duplications increased the number of *rxfp3*-like genes in teleosts to 7 (Figure 2 and Dataset S1: Tables S7, S8). Interestingly, our data mining uncovered that a few tetrapods retained *RXFP3-3*, but *RXFP3-2* appears to have been completely lost in the early tetrapod ancestor (Figure 2 and Dataset S1: Tables S4, S6). Using the available *RXFP3-3* sequences from opossum, cow and pig we located the *RXFP3-3* pseudogene in human and confirmed its common origin (*GAC “A4”*) with its medaka orthologs (Dataset S1: Table S1).

**Figure 3:**
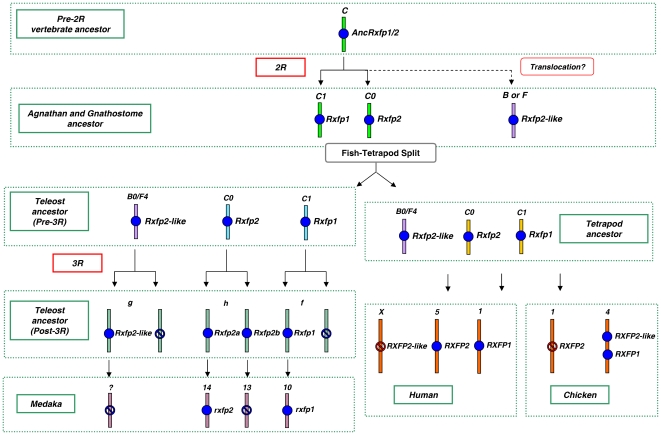
Reconstruction of the genetic events that led to the diversification of *RXFP1/2*-type receptor genes in vertebrates. Symbols and linkage group numbering same as in *Figure 2*.

Our tracing of the ancestral origins of *RXFP1* and *RXFP2* receptors in human and medaka showed that both of these genes originated from *VAC “C”* (Dataset S1: Table S1). Thus we concluded that 2R led to the duplication of an ancestral gene, *AncRxfp1/2*, of which only 2 orthologs (*RXFP1* and *RXFP2*) were retained in human and medaka (Figure 3 and also Figure S2). Interestingly, duplicates of *rxfp1* and *rxfp2* were also lost after 3R in stickleback (*Gasterosteus aculeatus*), tetraodon (*Tetraodon nigroviridis*) and fugu (*Takifugu rubripes*), but were partly retained in zebrafish, in which we found two *rxfp2* orthologs (Figure S4).

The two genes previously reported as *RXFP1* and *RXFP2* in chicken, turned out to have an evolutionary history that was slightly different from that of their counterparts in other vertebrates. Chicken “*RXFP1*” was traced to *GAC* “*C1*” (implying its orthology to the *RXFP1* of human and medaka), but the chicken “*RXFP2*” gene was traced to a different ancestral linkage group (*GAC “B0”* or “*F4*”) than the expected *GAC* “*C2*” (Dataset S1: Table S1). Further analyses confirmed that this gene does not share synteny with either *RXFP1* or *RXFP2* human genes and we therefore rename it *RXFP2-like*. Subsequently, we identified an ortholog of this *RXFP2-like* gene in some other vertebrates, such as zebrafish and opossum, and found a pseudogene of the *RXFP2-like* gene on human chromosome X next to *STARD8*, its neighbouring gene in chicken (Dataset S1: Table S6). Convincingly, BLASTn searches also revealed a pseudogene of *RXFP2* in the region of the chicken genome orthologous to that hosting *RXFP2* in other vertebrates. To incorporate this information about the evolutionary relationship among genes, here we adopt an origin-based nomenclature for the novel genes identified in this study but retain, as much as possible, the traditional naming scheme for the *RLN/INSL* and *RXFP* gene families (Table 1).

**Table 1:** Explanation of the nomenclature used for the hypothetical ancestral genes that gave rise to the three gene families discussed in this study (receptors *RXFP3/4, RXFP1/2* and their ligands *RLN/INSL*) via three rounds of WGD (*1R, 2R* and teleost-specific *3R*).

Origin	Gene Family: *RXFP3/4* (*SALPR/GPCR142*)							
*Pre-2R*	*AncRxfp3/4*							
*Post-1R paralogs*	*AncRxfp3-I*				*AncRxfp3-II*			
*Post-2R paralogs*	*RXFP3-1*		*RXFP3-2*		*RXFP3-3*		*RXFP3-4* *	
*Post-3R paralogs*	*rxfp3-1*	†	*rxfp3-2a*	*rxfp3-2b*	*rxfp3-3a* ‡	*rxfp3-3b*	*rxfp4*	†

Origin	Gene Family: *RXFP1/2 (LGR7/8)*					
*Pre-2R*	*AncRxfp1/2*					
*Post-1R paralogs*	*AncRxfp1*			*AncRxfp2*		
*Post-2R paralogs*	*RXFP1*		†	*RXFP2*		*RXFP2-like* **
*Post-3R paralogs*	*rxfp1*	†		*rxfp2a*	*rxfp2b*	

Origin	Gene Family: *RLN/INSL (Relaxin family peptides)*							
*Pre-2R*	*AncRln-like*							
*Post-1R paralogs*	*AncRln-I*				*AncRln-II*			
*Post-2R paralogs*	*RLN*		*INSL3*		*RLN3*		*INSL5*	
*Post-3R paralogs*	*rln*	†	*insl3*	†	*rln3a*	*rln3b*	*insl5a*	*insl5b*

*We show that the gene known as “*RXFP4*” is one of the three ohnologs of *RXFP3-1*, hence based on its origin it should be termed “*RXFP3-4*”;

**The origins of *RXFP2-like* (present in zebrafish, amphibians, birds, reptiles and marsupials) remain controversial, it is possible that *RXFP2-like* is a post-2R descendant of *AncRxfp2*, in which case it should be called “*RXFP2-2*”, while the ortholog of human *RXFP2* should be called “*RXFP2-1*”;

‡ *rxfp3-3a* was locally duplicated in the Post-3R ancestor of zebrafish, medaka, stickleback and pufferfishes (hence *rxfp3-3a* and *rxfp3-3b*); in zebrafish there are three paralogous *rxfp3-3a* genes: *3-3a1*, *3-3a2* and *3-3a3*;

† Gene loss

### Linkage relationships among *RLN/INSL* and *RXFP* genes have changed over evolutionary time

**Figure 4:**
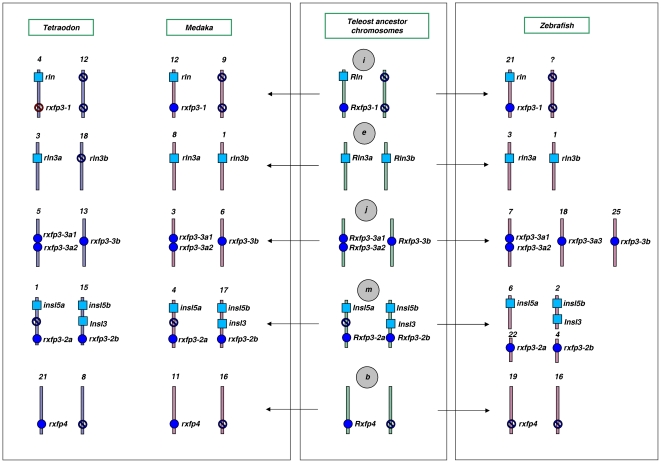
The evolution and genetic linkage of *RLN/INSL* (ligand) and *RXFP3/4* (receptor) loci in the pre-3R teleost ancestor and three species of teleost fish. Notice that among the three fish species analysed, medaka's genome and *rln/insl-rxfp* gene sets are the most preserved and resemble those of the teleost ancestor. Tetraodon experienced lineage-specific loss of two genes, *rln3b and rxfp3-1*, which may indicate their co-evolution as a ligand-receptor pair. The *rxfp4* gene in zebrafish seems to have been replaced with an extra (zebrafish-specific) copy of an *rxfp3-3* gene. The syntenic linkage between *rln* and *insl3* (ligand) and *rxfp3-1* and *rxfp3-2*(b) (receptor) genes has been conserved in all three teleosts since the post-2R ancestor (see Figure 2). Overall this scheme demonstrates that the rln/insl-rxfp system in teleosts has taken a slightly different, and seemingly more complicated, evolutionary pathway compared to other vertebrates. Chromosome numbers in extant species are shown as numbers and in the teleost ancestor as letters.

According to the “fission” model of *VAC “A”* evolution, in the pre-2R vertebrate ancestor, the receptor *AncRxfp3/4* gene was in the same linkage group as the ligand *AncRln-like*. Our reconstruction shows that two of the *RXFP3* 2R-ohnologs (*RXFP3-1* and *RXFP3-2*) were linked to *RLN* and *INSL3* (Figure 2), while the remaining ohnologs became unlinked. These ancestral genetic linkage relationships have mostly persisted in teleosts (Figure 4), but they have dynamically changed in tetrapods resulting in different combinations of linkage pairs such as *INSL5-RXFP4, RLN3-INSL3* and *RXFP1-RXFP2-like* to name a few (Figure S5).

We additionally used an ancestral genome reconstruction model by Putnam *et al*. [20] (the “P” model, see Figure 1 and Supporting Information S1), to address the ancestry of human *RLN/INSL/RXFP* genes. Consistent with the results obtained using the N-model, the P-model places all human *RLN/INSL* genes together with *RXFP3-1* in one ancestral linkage group. Likewise *RXFP1* and *RXFP2* were traced to one linkage group independent from the one harboring *RXFP2-like*. In disagreeement with the N-model, the ancestral linkage group predicted for *RXFP3-3* differs from that harboring *RXFP3-1*, and *RXFP4* could not be assigned to any ancestral region (Figure S3).

### RXFP phylogenetic reconstruction supports strong role of WGDs in gene duplication events

**Figure 5:**
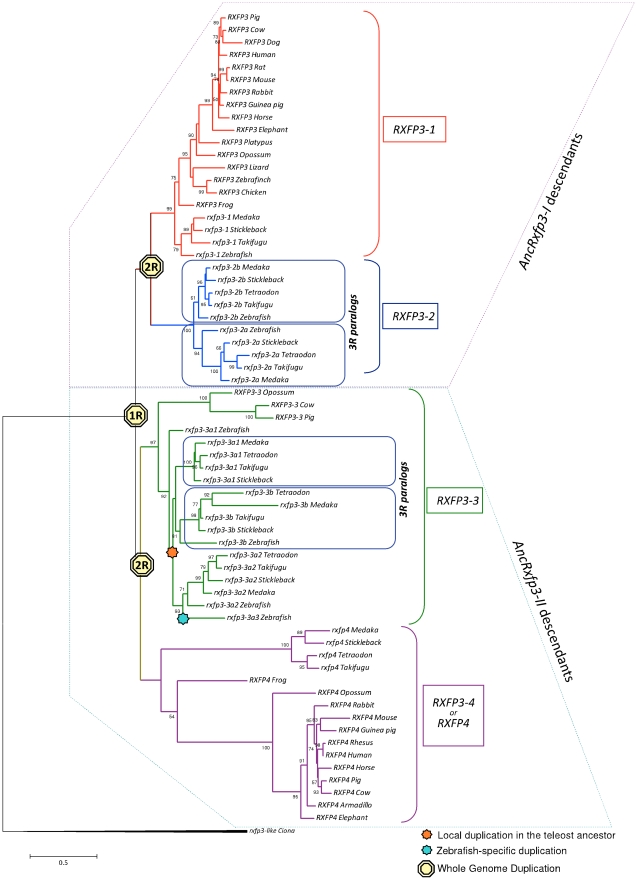
Phylogenetic reconstruction of the evolutionary relationship among vertebrate RXFP3/4 protein sequences. Reconstruction performed as outlined in methods with *G = 0.91* and *I = n/a*. Numbers at each node indicate the bootstrap values (only values exceeding 50% shown). Teleost rxfp3-2 underwent duplication yielding two 3R-paralogs, rxfp3-2a and rxfp3-2b, while teleostean ancestral rxfp3-3 was duplicated giving rise to typically three rxfp3-3 loci in modern teleosts: 3R generated rxfp3-3a and rxfp3-3b, while a local duplication generated rxfp3-3a1 and rxfp3-3a2. Solely in zebrafish, rxfp3-3a2 duplicated again giving rise to rxfp3-3a3, an event which appears to have occurred coincidently with the exclusive loss of rxfp4 in zebrafish.

The second goal of this study was to use other types of analysis, such as phylogeny and small-scale synteny, to corroborate the above model of evolution of the vertebrate RLN/INSL-RXFP systems in a broader range of vertebrates. We created a protein database and subsequently phylogenetic trees of RLN/INSL and RXFP-type genes for vertebrates and a few pre-2R diverging taxa, based on publicly annotated genes and included a few that we identified *de novo* (Dataset S1: Tables S4–S11). Overall, we find that the phylogenetic relationship of the receptor RXFP3/4 sequences clearly recapitulates their proposed WGD-driven origination: the proposed 1R-descendants cluster into two groups, AncRxfp3-I versus AncRxfp3-II, while the proposed 2R-descendants are sister clades, i.e. RXFP3-1/RXFP3-2 and RXFP3-3/RXFP3-4 as expected (Figure 5). Because most tetrapods lost half of their post-2R *RXFP3* ohnologs, the RXFP3-2 and RXFP3-3 clades mostly contain teleostean sequences.

**Figure 6:**
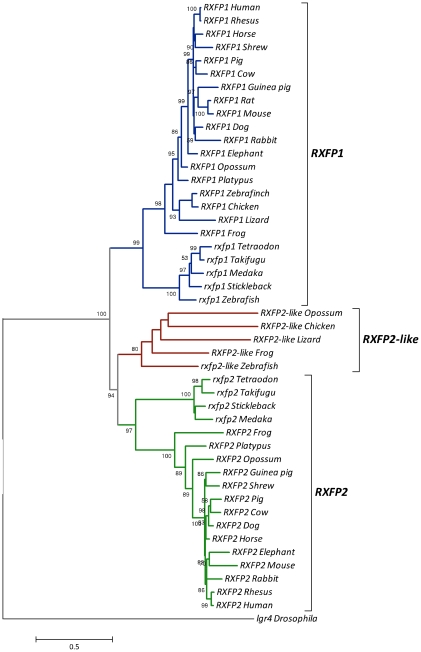
Phylogenetic reconstruction of the evolutionary relationship among vertebrate RXFP1/2 protein sequences. Phylogenetic tree reconstructed as outlined in methods with *G = 0.958* and *I = 0.034*. Numbers at each node indicate the bootstrap values (only values exceeding 50% shown). Due to their incomplete nature, not all sequences from our created database (see Methods) were included in this tree (e.g. zebrafish rxfp2a and rxfp2b and medaka rxfp2).

The RXFP1/2 phylogenetic tree (Figure 6) also generally supports the reconstruction model: there are 3 distinct clades for RXFP1, RXFP2 and RXFP2-like, and the RXFP2-like clade is sister to RXFP2, a clustering that supports the ohnologous nature of the relationship between *RXFP2-like* and *RXFP1/2* genes, rather than a pre-2R origin of *RXFP2-like*. To examine this more closely, we analyzed several vertebrate RXFP1/2 and RXFP2-like protein sequences together with invertebrate rxfp1/2-type proteins, and found that all vertebrate sequences clustered together (Figure S6), indicating that all 3 genes (i.e. *RXFP1*, *RXFP2* and *RXFP2-like*) originated after the divergence of protochordates.

## Discussion

Although it is now widely accepted that the two rounds of WGD that took place early in vertebrate evolution played a crucial role in the diversification of many vertebrate gene families [21], the processes by which WGD-driven gene family evolution occurred are not easy to determine. This has been shown to be true for the three vertebrate gene families encoding relaxin hormones and their receptors (*RLN/INSL, RXFP1/2* and *RXFP3/4*), whose duplication history and invertebrate origins we analyzed here. By combining information from ancestral genome reconstructions with phylogenetic and syntenic data, we were able to elucidate the origin of the *RLN/INSL* and *RXFP* genes. While the phylogenetic and syntenic data confirm the paralogous nature of the *RLN/INSL* and *RXFP* genes, the ancestral genome reconstructions strongly suggest that the four *RLN/INSL* and four *RXFP3/4*-type genes present in the gnathostome ancestor arose during 2R, and are thus ohnologs, and that two of the three *RXFP1/2* genes are similarly ohnologs, while the third *RXFP1/2*-like gene has an unclear origin. This is the first study to show the full evolutionary origin of the *RLN/INSL* and *RXFP* genes and the role of both 2R and 3R events in their diversification. Our study also supports the linkage of the ancestral *RLN/INSL* (ligand) and *RXFP3/4* (receptor) loci in the pre-2R vertebrate ancestor genome as first suggested by Olinski *et al*. [22], and at the same time reveals how these linkage relationships have been retained in some lineages while new linkage relationships have arisen in other lineages.

### The role of WGDs in the diversification of relaxin ligand-receptor systems

We based the reconstruction of the *RLN/INSL* and *RXFP* gene history principally on Nakatani *et al*.'s [12] model of vertebrate genome evolution. It has been proposed that the major vertebrate novelties, such as their structurally complex nervous, immune and reproductive systems, arose as a result of the massive amplification of genes that occurred during 2R [21], [23]. By making the necessary assumption that our focal genes remained in the given linkage groups since the pre-2R vertebrate ancestor, we deduced that the diversification of *RLN/INSL* and *RXFP* genes was coincidental with 2R events, suggesting that they probably played an important role in the establishment of neuroendocrine and reproductive regulation in early vertebrate evolution.

We also observe that the teleost-specific 3R, which strongly contributed to the genetic richness of teleosts and their biological success [24], [25], further increased the number of *rln/insl* and *rxfp* genes. However, in contrast to the 1R and 2R events, only those genes potentially involved in neuroendocrine regulation (*rln3*, *insl5* and half of the *rxfp3/4*-type receptors), but not reproduction (*rln*, *insl3* and *rxfp1/2*-type receptors) were retained after 3R in teleosts. The post-3R retention of *rln3* and *insl5* paralogs was paralleled by the retention of duplicates of *rxfp3-2* and *rxfp3-3* suggesting both co-functioning but also subfunctionalization of their neuroendocrine functions. Overall, we demonstrate that the large number of teleost receptor *rxfp3* genes is only partly attributable to teleost-specific duplications (which was proposed as the sole factor driving their diversification in a previous study [26]), but rather also resulted from the massive loss of *RXFP3* ohnologs in tetrapods.

### The “tripartite” origins of relaxin signaling

With respect to the origin of the RXFP receptors, we find that while both amphioxus and sea urchin genomes seem to be devoid of *rxfp3*-type genes [27], [28] and the two *rxfp3*-type genes in *C. intestinalis* are very divergent from their vertebrate analogs (Figure 5), early deuterostome lineages witnessed many lineage-specific expansions of the *RXFP1/2* locus (Figure S6). Intriguingly, three of the 5 amphioxus *rxfp1/2*-type genes appear orthologous to human *RXFP1* and *RXFP2* based on their shared ancestral chordate linkage groups as shown using the “P”-model (Figure S3 and Dataset S1: Table S3). Collectively, given the observation of multiple *rln/insl* and *ins/igf* [19], [22], [29], [30] and *rxfp1/2*-type genes, which are unmistakably evolutionarily related to their vertebrate counterparts, combined with the virtual absence of *rxfp3*-type genes in echinoderms and cephalochordates, we propose that the signaling of the ancestral RLN/INSL peptide in the chordate ancestor occurred via RXFP1/2-type receptors. Only at the onset of 2R, was the RXFP3/4-type receptor recruited to produce a signaling system encoded by 3 genes, composed of 2 receptors and a single ligand. It is tempting to hypothesize that this ancestral 2-receptor system had a dual function and played roles in both reproductive (using RXFP1/2-type receptor) and neuroendocrine processes (via RXFP3/4-type receptor).

The hypothesis that the *AncRln-like* locus exhibited co-expression in reproductive and neuroendocrine tissues and signaled via both RXFP1/2 and RXFP3/4 type receptors is supported by the following findings: 1) the dual functionality of human RLN3, considered to be the “oldest” member of the relaxin peptide family in vertebrates, a neuropeptide with the ability to trigger reproductive responses [31] and 2) the discovery of the relaxin-like nature of a starfish gonadotropin which is produced by the echinoderm's nervous system and directly influences the maturation of eggs in the ovary of starfish, probably via a GPCR receptor [32], [33]. This hypothesis also provides a rationale for the retention of the post-1R *Rln/Insl* duplicates and further helps to explain how the relaxin peptides have evolved to work with two divergent receptor types. If the neuroendocrine and reproductive actions of the pre-1R hormone were mediated by both RXFP1/2- and RXFP3/4-type receptors, then the post-1R loci (*AncRln-I* and *AncRln-II*) could have undergone subfunctionalization and specialization in terms of the tissues they targeted, i.e. either reproductive or neuroendocrine, and the receptors with which they functioned.

### Issues with currently used nomenclature

By thus elucidating the origin of genes, our model underscores the somewhat artificial nature of both ligand and receptor nomenclature. For ligands, we show that all *INSL* (insulin-like) genes independently originated from *RLN* (relaxin-like) genes (and not from an ancestral *INSL* gene, as previously hypothesized [17]). For receptors, currently only 4 *RXFP* genes (*RXFP1-4*) are recognized, those present in humans and some other placentals, while, in fact, there are seven *RXFP* genes of independent origin in vertebrates (three *RXFP1/2* and four *RXFP3/4*-type), at least six of which are ohnologs, and six of which are present in at least one copy in tetrapods. We also show that *RLN3* and *INSL3* are ohnologs, and not closely related genes that arose from a tandem duplication event as previously hypothesized [18]. Furthermore, all four *RLN/INSL* ohnologs were retained after 2R, which contradicts a less parsimonious scenario discussed by Hoffman and Opazo [17], in which one of *RLN/INSL* genes is lost in all vertebrates. Overall, our model for *RLN/INSL* evolution in vertebrates is consistent with the hypothesis postulating that *INSL3* and *RLN* evolved as a subfamily distinct from that formed by *RLN3* and *INSL5* [13]. Our model, however, dates the diversification of the two subfamilies back to the agnathan and gnathostome ancestors, while Wilkinson and Bathgate [13] refer it to the more recent appearance of mammals.

### Genetic linkage of RXFP receptors and their ligands

To the best of our knowledge, this is the first study to reveal the dynamic nature of the changing linkage relationships among relaxin family peptides and their receptor genes. The association of *RXFP3/4* genes with the *RLN/INSL* paralogon was first documented by Olinski *et al*. [22] and the linkage of human *INSL5* and *RXFP4* on one chromosome also mirrors their ligand-receptor interaction [34]. Nevertheless, we show that *INSL5* and *RXFP4* occupied different linkage groups in the gnathostome and tetrapod ancestors and only became linked in the eutherian ancestor (Figure 2). Chromosomal linkage of receptor and ligand genes, and other functionally linked or interacting genes, has been known for a number of unrelated gene families and is more common in the human genome than expected by chance [35], [36]. To explain this phenomenon, it has been proposed that receptor-ligand linkage could be advantageous for the creation of new receptor-ligand pairs when they result from block duplications [35]. However, this beneficial effect of linkage would not pertain to genes duplicated via WGDs, as is the case of *RLN/INSL* and *RXFP* loci. Instead, we propose that the ancestral *RXFP3/4*-*RLN/INSL* linkage was fortuitous and only the subsequent retention of linkage in descendants may have been subject to selection for an advantageous topology, which may have caused the co-expression of ligands and receptors thereby increasing the frequency of their interaction. Although the original linkage was disrupted for one of the post-1R ohnolog pairs, linkage of certain *RLN/INSL-RXFP3* pairs has been conserved in some organisms, e.g. in medaka, while not in others, such as in rat (Figure 3 and Figure S6). In this regard, it is interesting that the chromosomal sections harboring the *INSL/RLN* paralogons contain many other conserved gene families, such as the major histocompatibility complex genes, whose origins are traceable to singular pre-2R ancestor genes [21]. This suggests that conservation of the linkage relationship among the *RLN/INSL* and *RXFP* genes may result from conservation of synteny at a larger scale. At the same time, vertebrates have also acquired novel and lineage-specific gene linkages, such as that of *RLN3-INSL3* in opossum, human and pig and *RXFP1-RXFP2-like* in chicken (Figure S6), which could be explained by other factors such as recurrent evolutionary chromosomal breaks in the fragile parts of genomes containing these genes [37].

### Difficulties encountered using paleogenomics models

Finally, it should be noted that there can be difficulties in resolving the origin of some genes using ancestral genome reconstructions. This was observed here for the *RXFP2-like* gene. When a gene maps to a different ancestral linkage group than expected (*VAC “F”* versus *“C”* in the case of *RXFP2-like*), it is difficult to determine if 1) this gene has independent origins from its expected ohnologs or 2) it underwent a single-gene translocation that caused it to move from its authentic chromosomal fragment after duplication or whether 3) the ancestral linkage groups are inaccurately reconstructed. For *RXFP2-like* it seems possible that there were 2 genes present in the ancestral pre-2R genome, because there are many *rxfp1/2*-type genes in primitive chordates. However, the *RXFP2-like* genes from vertebrates cluster closely within the vertebrate *RXFP1/2* sequences (Figure S6), suggesting that *RXFP2-like* is an ohnolog of *RXFP1* and *RXFP2* genes. Hence *RXFP2-like* was probably either translocated from its authentic position in the gnathostome ancestor or we are confronted with an inaccurate reconstruction of *VAC “C”* and *“F”*. Interestingly, there is a documented example of a single gene translocation of a duplicated insulin (*INS*) gene in rodents [38]. When traced using the N-model (not shown here) the murid-specific *INS* gene maps to an ancestral linkage group different from the expected *VAC “D”* owing to a single gene-translocation that took place early in the evolution of mice and rats [38].

### Conclusions

In summary, the origination of relaxin hormones and their two distinct classes of receptors in vertebrates was strongly driven by whole genome duplication events. We postulate that the relaxin hormone-receptor signaling system in the pre-2R ancestor consisted of three components, one ligand and two receptors, and had a dual (reproductive and neuroendocrine regulatory) function. The genetic linkage of *RLN/INSL* and *RXFP3/4* genes, which has been highly conserved in teleosts since the post-2R ancestor, probably played a role in the original establishment of ligand-receptor interactions between ancestral RLN/INSL and RXFP3/4 proteins. We show that most of the ligand and receptor genes duplicated during 2R (or 3R) and that, compared to tetrapods, teleosts have had significantly higher post-2R retention rates of *RXFP* genes. Our findings about the evolution of relaxin hormones and their receptors should facilitate further research on this system in various vertebrates, including both placental and non-placental taxa. For instance, the discussed 2R-driven model of evolution should raise questions about the number of involved genes in early diverging vertebrates, such as jawless fish, whose status in relation to 2R has until recently been debated [21].

Overall, our study highlights the utility of incorporating ancestral genome data into investigations of the origin, linkage relationship and duplication history of gene families. The methodology employed here will hopefully be useful in similar studies, where traditional approaches may fail to clearly resolve the origin of genes due to their small size, strong roles of selection or insufficient synteny data. Presently, however, a major drawback of the method is the absence of a unified scheme, which would avoid having to perform the time consuming and tedious manual inspection of multiple ancestral genome reconstruction models. In the future this problem could be resolved by designing appropriate computer software. Thus, rather than being viewed as a primarily heuristic tool for studying large scale genome evolution, ancestral genome reconstructions have a potential to form the basis of an instrument that could be routinely consulted to supplement traditional bioinformatic analyses.

## Methods

### Tracing of the duplication history of *RLN/INSL*, *INS/IGF* and *RXFP* genes

Detailed methods used to trace the evolutionary history of genes are provided in Supporting Information S1. A brief overview of the procedure is given here: First, using their exact map positions, we mapped the *RLN/INSL, RXFP* and *INS/IGF* genes found in human, medaka and chicken to their corresponding chromosomal segments. These chromosomal segments were then matched to the linkage groups in ancestral genomes primarily according to Nakatani *et al*.'s [12] model, but we also invoked other vertebrate genome reconstructions [39], [40], in particular Putnam et al's [20], as needed. Finally we compared the results obtained for each of the three taxa to resolve the positions of the focal genes at consecutive stages of the vertebrate genome evolution. Where discrepancies arose and the genes reported as “orthologous” were traced to different ancestral linkage groups, we performed small-scale synteny analyses (details below) to clarify the relationship of individual genes among taxa.

### Identification of *RLN/INSL* and *RXFP(-like)* sequences across vertebrates

All annotated *RLN/INSL* and *RXFP* coding sequences with their genomic positions were retrieved from the Ensembl v.60 database (http://ensembl.org) for 13 mammals (11 placentals, opossum and platypus), 3 reptiles (anole lizard, chicken and zebrafinch), 2 amphibians (clawed frog and edible frog) and 5 teleosts (Dataset S1: Tables S4–S8). The annotated sequences for rhesus monkey were obtained from NCBI (http://ncbi.nlm.nih.gov/gene). For *RXFP1/2* genes, when multiple splice variants were available, the longer variant was chosen, unless shorter variants had been confirmed to be functional.

Using the more or less complete sets of human, mouse, zebrafish and medaka sequences as reference, we performed searches of the databases at both Ensembl and NCBI to look for unannotated and/or yet unidentified genes in other tetrapods and teleosts using the NCBI BLAST package [41]. Additionally, to either confirm the identity of sequences obtained using the above procedure or to search for other difficult to identify genes, we searched the paralogons syntenic to previously determined human/teleost *RLN/INSL* [16] or *RXFP* genes in Ensembl by using the Genscan tool or the MIT Genscan server (http://genes.mit.edu/GENSCAN.html) in combination with the conserved-domain search tool (http://ncbi.nlm.nih.gov/Structure/cdd/wrpsb.cgi), or by blasting the entire syntenic regions via BLASTn in NCBI with an appropriate query. The synteny analysis for the *RXFP* genes was done using either the Genomicus v.60.01 server (http://dyogen.ens.fr/genomicus-60.01/cgi-bin/search.pl), the appropriate Ensembl tools and/or manual identification of orthologous regions through subjecting genes to BLASTp at NCBI.

### *RXFP*-type genes in pre-2R taxa

For *C. intestinalis*, 8 *rxfp1/2*-type genes were retrieved from Ensembl and 2 candidate *rxfp3/4*-type genes were obtained from ANISEED (http://crfb.univ-mrs.fr/aniseed). Five amphioxus *rxfp1/2*-type and were retrieved from GenBank. The 6 *ilp* genes from the amphioxus database were previously analyzed and shown to have syntenically shared genes with the vertebrate Insulin-Relaxin loci [29]. Our searches in the sea urchin database (http://www.spbase.org/SpBase/) yielded 27 *rxfp1/2*-like sequences. 2 *rxfp1/2*-like genes (*lgr3* and *lgr4*) were obtained from Ensembl Metazoa (http://metazoa.ensembl.org) for fruit fly (*Drosophila melanogaster*).

### Phylogenetic reconstruction of the relationships among *RXFP* genes

The alignment of *RXFP* sequences (available upon request) was accomplished using MUSCLE [42] as implemented in MEGA v. 5.01 [43] and through manual adjustments. Phylogenetic reconstruction of protein sequences was carried out in Phyml [44] using: for *RXFP* genes, the LG model of sequence evolution and with estimated or fixed values for *G*, the shape parameter for the gamma distribution, and *I*, the proportion of invariant sites, depending on what was determined to be the best model of amino acid sequence evolution based on AIC as implemented in ProtTest [45]. Confidence in the phylogenetic reconstruction was assessed using 1000 replicate bootstrap samples. The phylogenetic relationship among invertebrate *rxfp1/2*-type genes was reconstructed separately following the same method.

## Supporting Information

Figure S1 **The alternative (“fusion”) scenario of duplication and rearrangement history for VAC “A” according to the N-model.**
(PDF)

Figure S2 **Two alternative scenarios for the 2R-driven duplication of the AncRxfp1/2 gene.**
(PDF)

Figure S3 **Comparison of the results obtained using two ancestral genome reconstructions.** Top: Tracing of human RLN/INSL and RXFP-like genes in chordate linkage groups (CLG) using P-model; bottom: Tracing of human RLN/INSL and RXFP genes in pre-2R vertebrate ancestor chromosomes.
(PDF)

Figure S4 **Chromosomal location of rxfp1/2-type genes in three species of teleosts and in the post-3R teleost ancestor.**
(PDF)

Figure S5 **Dynamic changes in the chromosomal linkage relationships of RLN/INSLRXFP genes in tetrapods.**
(PDF)

Figure S6 **Phylogenetic reconstruction of RXFP1/2 proteins from vertebrates, protochordates and an echinoderm.**
(PDF)

Supporting Information S1 **The detailed methods explaining the use of ancestral genome reconstructions to trace the evolutionary history of individual focal genes.**
(PDF)

Dataset S1 **All accession numbers and map locations for genes used in this study (includes Tables S1–S12).**
(PDF)

## References

[1] Abi-Rached L, Gilles A, Shiina T, Pontarotti P, Inoko H (2002). Evidence of en bloc duplication in vertebrate genomes.. Nat Genet.

[2] Dehal P, Boore JL (2005). Two rounds of whole genome duplication in the ancestral vertebrate.. PLoS Biol.

[3] Jaillon O, Aury JM, Brunet F, Petit JL, Stange-Thomann N (2004). Genome duplication in the teleost fish Tetraodon nigroviridis reveals the early vertebrate proto-karyotype.. Nature.

[4] Kuraku S, Meyer A, Kuratani S (2009). Timing of genome duplications relative to the origin of the vertebrates: did cyclostomes diverge before or after?. Mol Biol Evol.

[5] Ohno S (1970). Evolution by gene duplication. Berlin, New York: Springer-Verlag.

[6] Taylor JS, Raes J (2004). Duplication and divergence: the evolution of new genes and old ideas.. Annu Rev Genet.

[7] Nei M, Gu X, Sitnikova T (1997). Evolution by the birth-and-death process in multigene families of the vertebrate immune system.. Proc Natl Acad Sci U S A.

[8] Wolfe KH (2001). Yesterday's polyploids and the mystery of diploidization.. Nat Rev Genet.

[9] Maere S, De Bodt S, Raes J, Casneuf T, Van Montagu M (2005). Modeling gene and genome duplications in eukaryotes.. Proc Natl Acad Sci U S A.

[10] Makino T, Hokamp K, McLysaght A (2009). The complex relationship of gene duplication and essentiality. Trends in Genet..

[11] Muffato M, Roest Crollius H (2008). Paleogenomics in vertebrates, or the recovery of lost genomes from the mist of time.. Bioessays.

[12] Nakatani Y, Takeda H, Kohara Y, Morishita S (2007). Reconstruction of the vertebrate ancestral genome reveals dynamic genome reorganization in early vertebrates.. Genome Res.

[13] Wilkinson TN, Bathgate RA (2007). The evolution of the relaxin peptide family and their receptors.. Adv Exp Med Biol.

[14] Halls ML, van der Westhuizen ET, Bathgate RA, Summers RJ (2007). Relaxin family peptide receptors--former orphans reunite with their parent ligands to activate multiple signalling pathways.. Br J Pharmacol.

[15] Gloriam DE, Foord SM, Blaney FE, Garland SL (2009). Definition of the G protein-coupled receptor transmembrane bundle binding pocket and calculation of receptor similarities for drug design.. J Med Chem.

[16] Good-Avila SV, Yegorov S, Harron S, Bogerd J, Glen P (2009). Relaxin gene family in teleosts: phylogeny, syntenic mapping, selective constraint, and expression analysis.. BMC Evol Biol.

[17] Hoffmann FG, Opazo JC (2011). Evolution of the relaxin/insulin-like gene family in placental mammals: implications for its early evolution.. J Mol Evol.

[18] Park JI, Semyonov J, Chang CL, Yi W, Warren W (2008). Origin of INSL3-mediated testicular descent in therian mammals.. Genome Res.

[19] Olinski RP, Dahlberg C, Thorndyke M, Hallbook F (2006). Three insulin-relaxin-like genes in Ciona intestinalis.. Peptides.

[20] Putnam NH, Butts T, Ferrier DE, Furlong RF, Hellsten U (2008). The amphioxus genome and the evolution of the chordate karyotype.. Nature.

[21] Kasahara M (2007). The 2R hypothesis: an update.. Curr Opin Immunol.

[22] Olinski RP, Lundin LG, Hallbook F (2006). Conserved synteny between the Ciona genome and human paralogons identifies large duplication events in the molecular evolution of the insulin-relaxin gene family.. Mol Biol Evol.

[23] Huminiecki L, Heldin CH (2010). 2R and remodeling of vertebrate signal transduction engine.. BMC Biol.

[24] Hoegg S, Brinkmann H, Taylor JS, Meyer A (2004). Phylogenetic timing of the fish-specific genome duplication correlates with the diversification of teleost fish.. J Mol Evol.

[25] Meyer A, Van de Peer Y (2005). From 2R to 3R: evidence for a fish-specific genome duplication (FSGD).. Bioessays.

[26] Wilkinson TN, Speed TP, Tregear GW, Bathgate RA (2005). Evolution of the relaxin-like peptide family.. BMC Evol Biol.

[27] Nordstrom KJ, Fredriksson R, Schioth HB (2008). The amphioxus (Branchiostoma floridae) genome contains a highly diversified set of G protein-coupled receptors.. BMC Evol Biol.

[28] Sodergren E, Weinstock GM, Davidson EH, Cameron RA, Gibbs RA (2006). The genome of the sea urchin Strongylocentrotus purpuratus.. Science.

[29] Holland LZ, Albalat R, Azumi K, Benito-Gutierrez E, Blow MJ (2008). The amphioxus genome illuminates vertebrate origins and cephalochordate biology.. Genome Res.

[30] McRory JE, Sherwood NM (1997). Ancient divergence of insulin and insulin-like growth factor.. DNA Cell Biol.

[31] McGowan BM, Stanley SA, Donovan J, Thompson EL, Patterson M (2008). Relaxin-3 stimulates the hypothalamic-pituitary-gonadal axis.. Am J Physiol Endocrinol Metab.

[32] Mita M, Yamamoto K, Nagahama Y (2011). Interaction of Relaxin-Like Gonad-Stimulating Substance with Ovarian Follicle Cells of the Starfish Asterina pectinifera.. Zoolog Sci.

[33] Mita M, Yoshikuni M, Ohno K, Shibata Y, Paul-Prasanth B (2009). A relaxin-like peptide purified from radial nerves induces oocyte maturation and ovulation in the starfish, Asterina pectinifera.. Proc Natl Acad Sci U S A.

[34] Liu C, Kuei C, Sutton S, Chen J, Bonaventure P (2005). INSL5 is a high affinity specific agonist for GPCR142 (GPR100).. J Biol Chem.

[35] Hurst LD, Lercher MJ (2005). Unusual linkage patterns of ligands and their cognate receptors indicate a novel reason for non-random gene order in the human genome.. BMC Evol Biol.

[36] Makino T, McLysaght A (2008). Interacting Gene Clusters and the Evolution of the Vertebrate Immune System.. Molecular Biology and Evolution.

[37] Bailey JA, Baertsch R, Kent WJ, Haussler D, Eichler EE (2004). Hotspots of mammalian chromosomal evolution.. Genome Biol.

[38] Shiao MS, Liao BY, Long M, Yu HT (2008). Adaptive evolution of the insulin two-gene system in mouse.. Genetics.

[39] Kasahara M, Naruse K, Sasaki S, Nakatani Y, Qu W (2007). The medaka draft genome and insights into vertebrate genome evolution.. Nature.

[40] Kemkemer C, Kohn M, Cooper DN, Froenicke L, Hogel J (2009). Gene synteny comparisons between different vertebrates provide new insights into breakage and fusion events during mammalian karyotype evolution.. BMC Evol Biol.

[41] Altschul SF, Madden TL, Schaffer AA, Zhang J, Zhang Z (1997). Gapped BLAST and PSI-BLAST: a new generation of protein database search programs.. Nucleic Acids Res.

[42] Edgar RC (2004). MUSCLE: multiple sequence alignment with high accuracy and high throughput.. Nucleic Acids Res.

[43] Tamura K, Peterson D, Peterson N, Stecher G, Nei M (2011). MEGA5: molecular evolutionary genetics analysis using maximum likelihood, evolutionary distance, and maximum parsimony methods.. Mol Biol Evol.

[44] Guindon S, Dufayard JF, Lefort V, Anisimova M, Hordijk W (2010). New algorithms and methods to estimate maximum-likelihood phylogenies: assessing the performance of PhyML 3.0.. Syst Biol.

[45] Abascal F, Zardoya R, Posada D (2005). ProtTest: selection of best-fit models of protein evolution.. Bioinformatics.

