# Supplementary material for: Using Paleogenomics to Study the Evolution of Gene Families: Origin and Duplication History of the Relaxin Family Hormones and Their Receptors
Source: PLoS One. 2012 Mar 21;7(3):e32923. doi: 10.1371/journal.pone.0032923 (PMC3310001; doi:10.1371/journal.pone.0032923)
Supplement: Figure S1 — The alternative (“fusion”) scenario of duplication and rearrangement history for VAC “A” according to the N-model. (PDF) [file pone.0032923.s001.pdf]

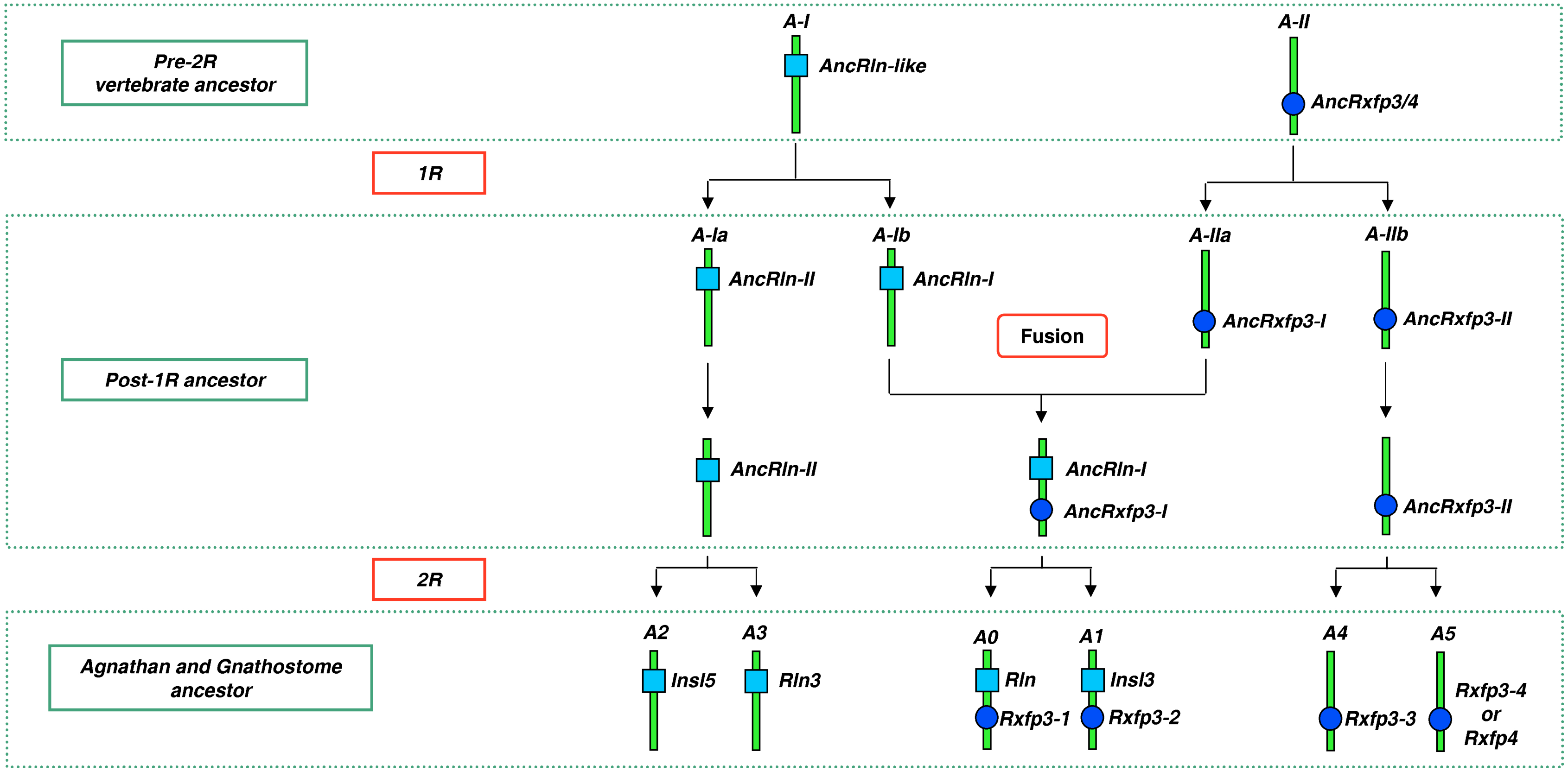

Figure S1. The alternative ("fusion") scenario of duplication and rearrangement history for *VAC "A"* according to the N-model. While the number and identity of post-2R daughter chromosomes in both scenarios (see Figure 2 in main text for the other scenario) is the same, the introduction of a *fusion* event (red box) and elimination of a *fission* event (red boxed in Figure 2, main text) in this scenario results in two pre-2R chromosomes (*A-I* and *A-II*) each carrying one gene (a ligand gene on one, and a receptor gene on the other). Notice that according to this scenario *AncRln-like* was not syntenically linked to *AncRxfp3/4* in the pre-2R vertebrate ancestor
