# Supplementary material for: Using Paleogenomics to Study the Evolution of Gene Families: Origin and Duplication History of the Relaxin Family Hormones and Their Receptors
Source: PLoS One. 2012 Mar 21;7(3):e32923. doi: 10.1371/journal.pone.0032923 (PMC3310001; doi:10.1371/journal.pone.0032923)
Supplement: Figure S2 — Two alternative scenarios for the 2R-driven duplication of the AncRxfp1/2 gene. (PDF) [file pone.0032923.s002.pdf]

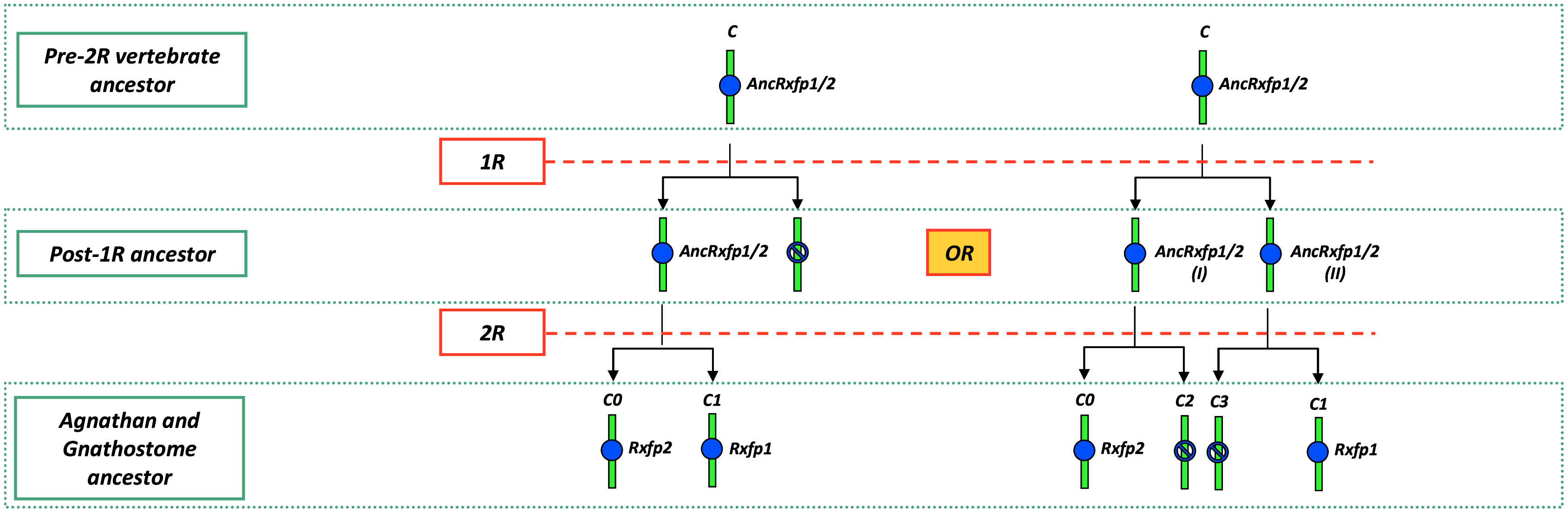

Figure S2. Two alternative scenarios for the 2R-driven duplication of the *AncRxfp1/2* gene. Note that based on the phylogenetic evidence (Figure 4b, main text), *RXFP2-like* is the paralog of *RXFP2*, in which case the two genes may have arisen as a result of 2R in *CLGs "C0"* and *"C2"*. The scenario on the right was hence adopted in the main text as more explanatory.
