# Supplementary material for: Using Paleogenomics to Study the Evolution of Gene Families: Origin and Duplication History of the Relaxin Family Hormones and Their Receptors
Source: PLoS One. 2012 Mar 21;7(3):e32923. doi: 10.1371/journal.pone.0032923 (PMC3310001; doi:10.1371/journal.pone.0032923)
Supplement: Figure S3 — Comparison of the results obtained using two ancestral genome reconstructions. Top: Tracing of human RLN/INSL and RXFP-like genes in chordate linkage groups (CLG) using P-model; bottom: Tracing of human RLN/INSL and RXFP genes in pre-2R vertebrate ancestor chromosomes. (PDF) [file pone.0032923.s003.pdf]

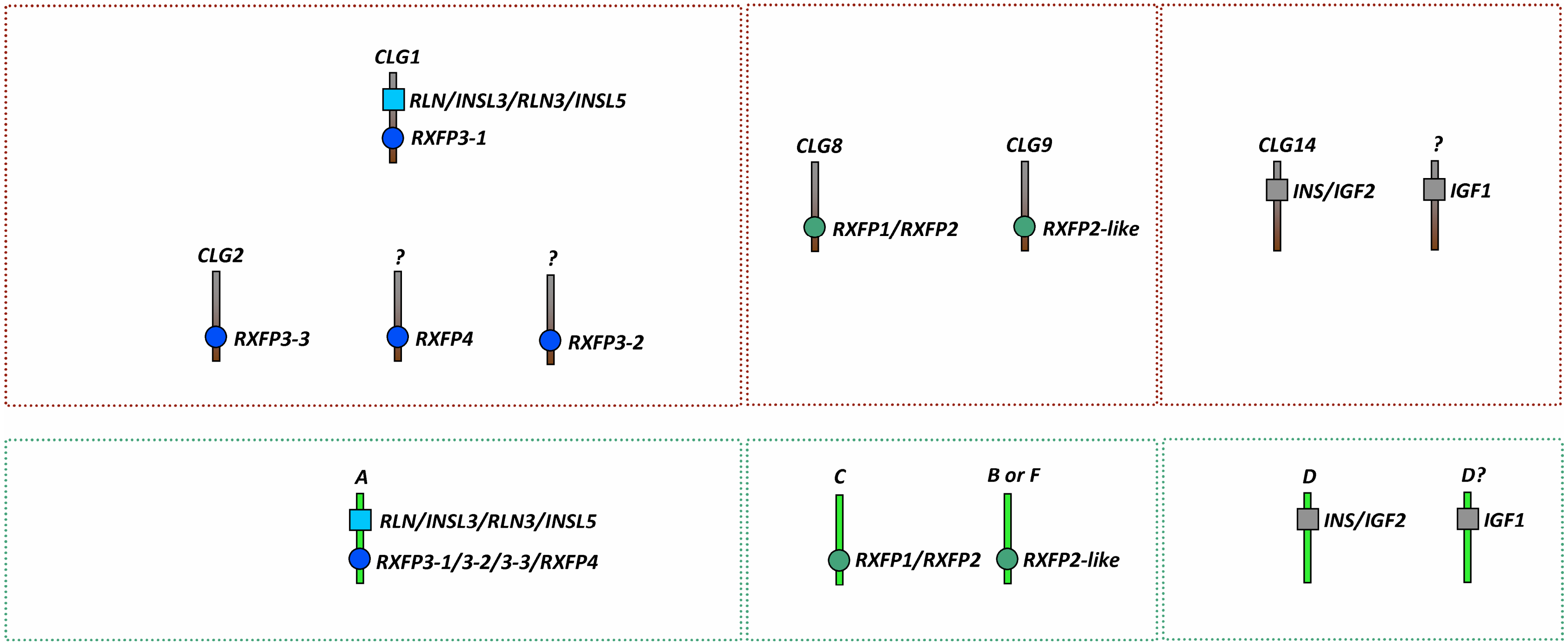

Figure S3. Comparison of the results obtained using two ancestral genome reconstructions **top:** Tracing of human *RLN/INSL* and *RXFP*-like genes in chordate linkage groups (*CLG*) using P-model; **bottom**: Tracing of human *RLN/INSL* and *RXFP* genes in pre-2R vertebrate ancestor chromosomes using N-model.
