# Supplementary material for: Using Paleogenomics to Study the Evolution of Gene Families: Origin and Duplication History of the Relaxin Family Hormones and Their Receptors
Source: PLoS One. 2012 Mar 21;7(3):e32923. doi: 10.1371/journal.pone.0032923 (PMC3310001; doi:10.1371/journal.pone.0032923)
Supplement: Figure S4 — Chromosomal location of rxfp1/2-type genes in three species of teleosts and in the post-3R teleost ancestor. (PDF) [file pone.0032923.s004.pdf]

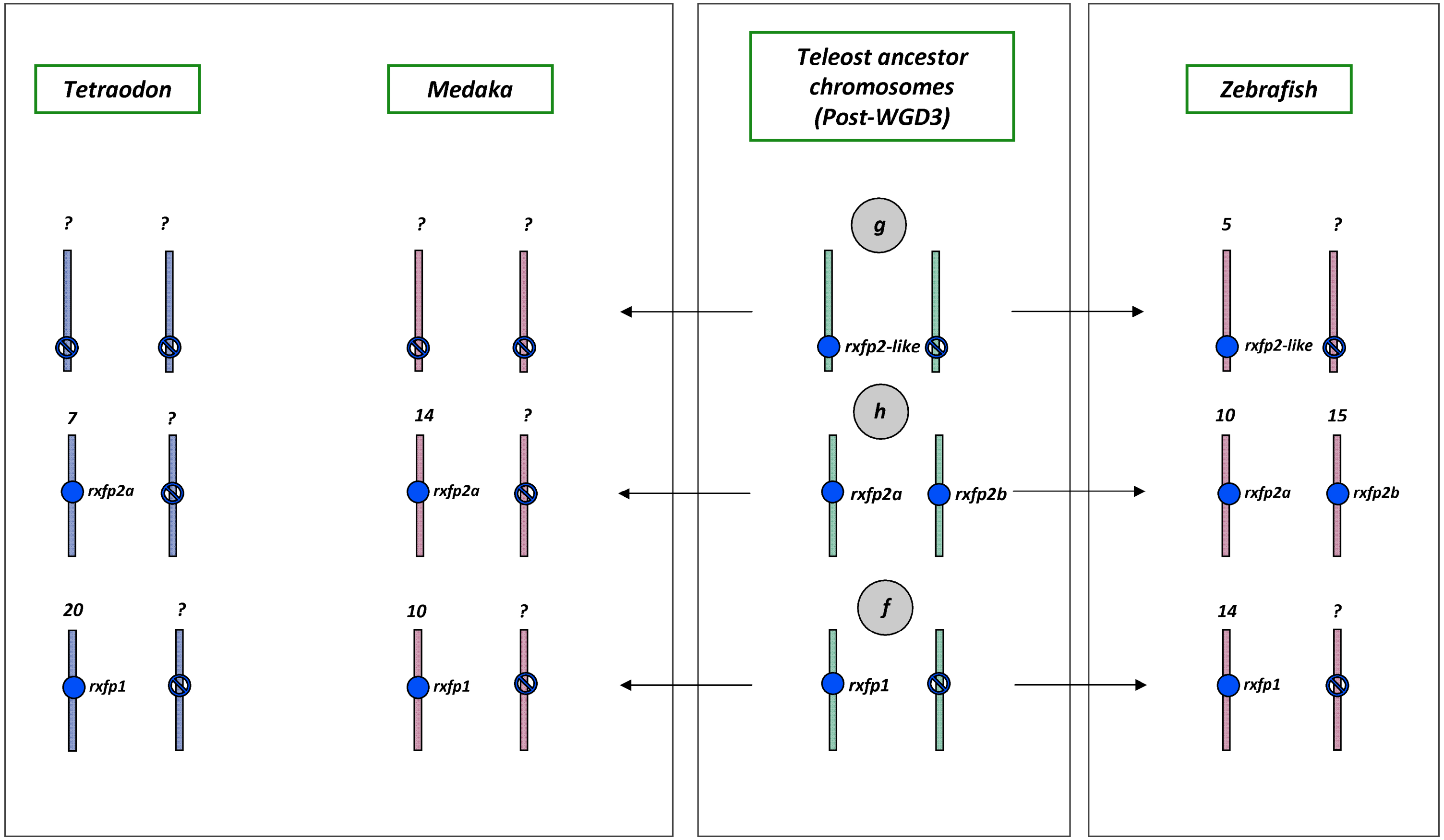

Figure S4. Chromosomal location of *rxfp1/2*-type genes in three species of teleosts and in the post-3R teleost ancestor
