# Supplementary material for: Using Paleogenomics to Study the Evolution of Gene Families: Origin and Duplication History of the Relaxin Family Hormones and Their Receptors
Source: PLoS One. 2012 Mar 21;7(3):e32923. doi: 10.1371/journal.pone.0032923 (PMC3310001; doi:10.1371/journal.pone.0032923)
Supplement: Figure S5 — Dynamic changes in the chromosomal linkage relationships of RLN/INSLRXFP genes in tetrapods. (PDF) [file pone.0032923.s005.pdf]

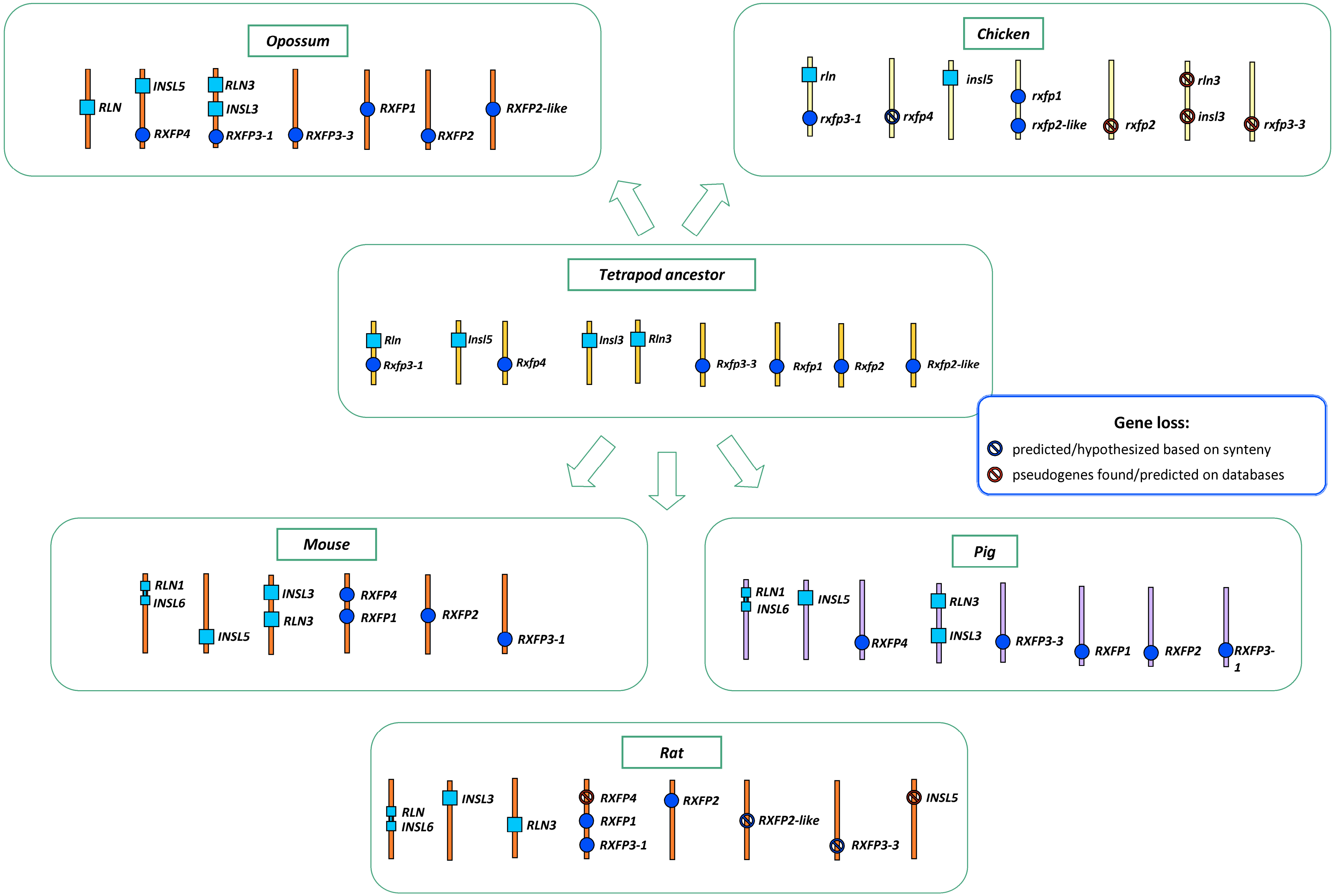
Opossum
RLN
INSL5
RXFP4
RLN3
INSL3
RXFP3-1
RXFP3-3
RXFP1
RXFP2
RXFP2-like
Chicken
rln
rxfp3-1
rxfp4
insl5
rxfp1
rxfp2-like
rxfp2
rln3
insl3
rxfp3-3
Tetrapod ancestor
Rln
Rxfp3-1
Insl5
Rxfp4
Insl3
Rln3
Rxfp3-3
Rxfp1
Rxfp2
Rxfp2-like
Gene loss:
predicted/hypothesized based on synteny
pseudogenes found/predicted on databases
Mouse
RLN1
INSL6
INSL5
INSL3
RLN3
RXFP4
RXFP1
RXFP2
RXFP3-1
Pig
RLN1
INSL6
INSL5
RXFP4
RLN3
INSL3
RXFP3-3
RXFP1
RXFP2
RXFP3-1
Rat
RLN
INSL6
INSL3
RLN3
RXFP4
RXFP1
RXFP3-1
RXFP2
RXFP2-like
RXFP3-3
INSL5

Figure S5.

Figure S5. Dynamic changes in the chromosomal linkage relationships of *RLN/INSL-RXFP* genes in tetrapods. Each bar represents a chromosome (IDs not shown for simplicity). Symbols and linkage numbering are as in *Figure 2* in main text.
