# Supplementary material for: Using Paleogenomics to Study the Evolution of Gene Families: Origin and Duplication History of the Relaxin Family Hormones and Their Receptors
Source: PLoS One. 2012 Mar 21;7(3):e32923. doi: 10.1371/journal.pone.0032923 (PMC3310001; doi:10.1371/journal.pone.0032923)
Supplement: Figure S6 — Phylogenetic reconstruction of RXFP1/2 proteins from vertebrates, protochordates and an echinoderm. (PDF) [file pone.0032923.s006.pdf]

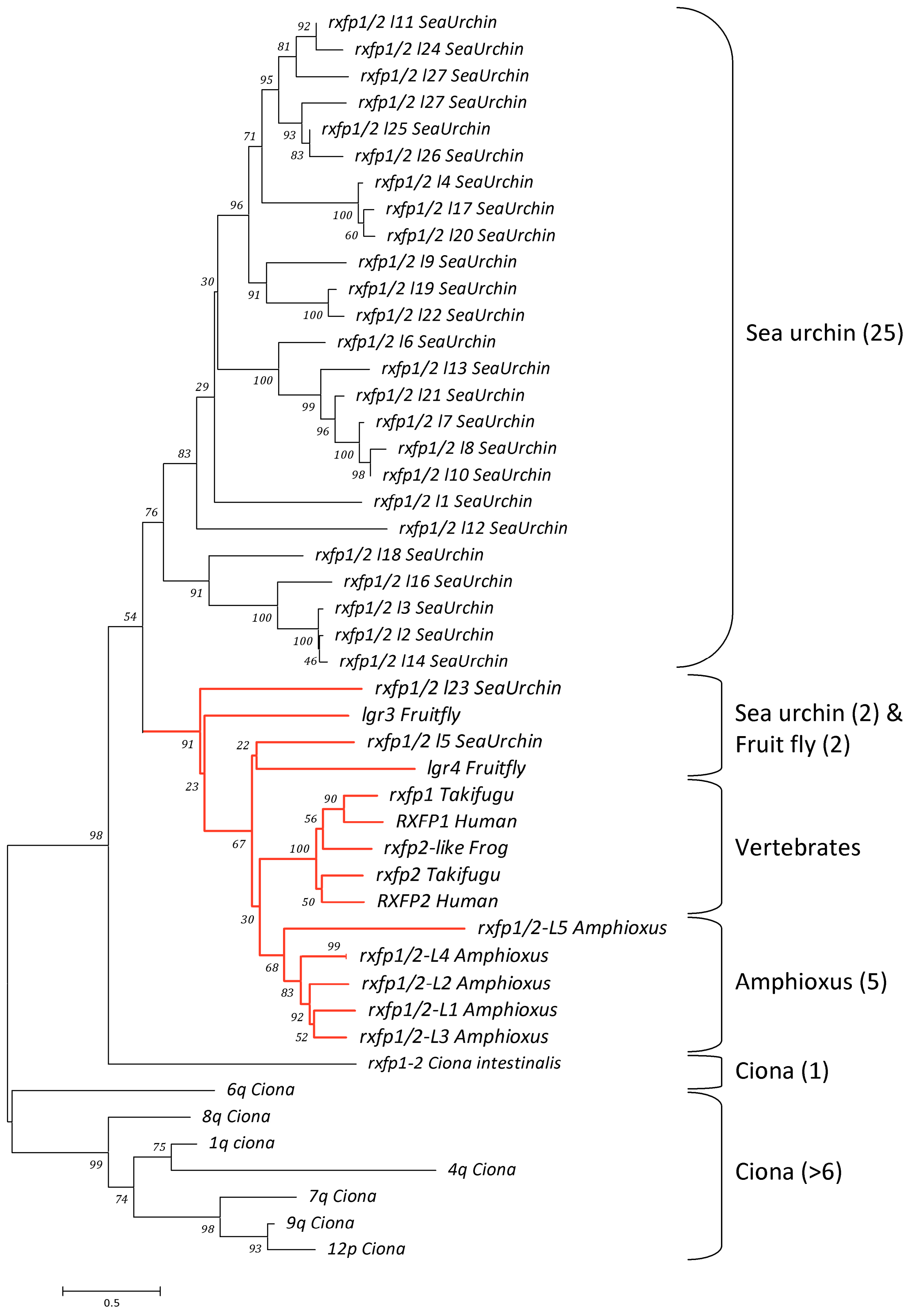
rxfp1/2 l11 SeaUrchin
rxfp1/2 l24 SeaUrchin
rxfp1/2 l27 SeaUrchin
rxfp1/2 l27 SeaUrchin
rxfp1/2 l25 SeaUrchin
rxfp1/2 l26 SeaUrchin
rxfp1/2 l4 SeaUrchin
rxfp1/2 l17 SeaUrchin
rxfp1/2 l20 SeaUrchin
rxfp1/2 l9 SeaUrchin
rxfp1/2 l19 SeaUrchin
rxfp1/2 l22 SeaUrchin
rxfp1/2 l6 SeaUrchin
rxfp1/2 l13 SeaUrchin
rxfp1/2 l21 SeaUrchin
rxfp1/2 l7 SeaUrchin
rxfp1/2 l8 SeaUrchin
rxfp1/2 l10 SeaUrchin
rxfp1/2 l1 SeaUrchin
rxfp1/2 l12 SeaUrchin
rxfp1/2 l18 SeaUrchin
rxfp1/2 l16 SeaUrchin
rxfp1/2 l3 SeaUrchin
rxfp1/2 l2 SeaUrchin
rxfp1/2 l14 SeaUrchin
rxfp1/2 l23 SeaUrchin
lgr3 Fruitfly
rxfp1/2 l5 SeaUrchin
lgr4 Fruitfly
rxfp1 Takifugu
RXFP1 Human
rxfp2-like Frog
rxfp2 Takifugu
RXFP2 Human
rxfp1/2-L5 Amphioxus
rxfp1/2-L4 Amphioxus
rxfp1/2-L2 Amphioxus
rxfp1/2-L1 Amphioxus
rxfp1/2-L3 Amphioxus
rxfp1-2 Ciona intestinalis
6q Ciona
8q Ciona
1q ciona
4q Ciona
7q Ciona
9q Ciona
12p Ciona
Sea urchin (25)
Sea urchin (2) &
Fruit fly (2)
Vertebrates
Amphioxus (5)
Ciona (1)
Ciona (>6)
0.5

Figure S6.

Figure S6. Phylogenetic reconstruction of RXFP1/2 proteins from vertebrates, protochordates and an echinoderm. All of the amphioxus rxfp1/2-like proteins cluster closely to vertebrate RXFP1/2's and RXFP2-like sequences, while two of the 27 sea urchin rxfp1/2-like proteins are found in a clade with fruit fly lgr3 and lgr4 in another sister clade to RXFP1/2-like genes. The Ciona rxfp1/2-like genes appear distantly related to the entire protostome-deuterostome RXFP1/2 cluster.
Fruit fly lgr3 (FBgn0039354) and lgr4 (FBgn0085440) genes were obtained from Ensembl Metazoa (http://www.metazoa.ensembl.org).
