# Supplementary material for: Using Paleogenomics to Study the Evolution of Gene Families: Origin and Duplication History of the Relaxin Family Hormones and Their Receptors
Source: PLoS One. 2012 Mar 21;7(3):e32923. doi: 10.1371/journal.pone.0032923 (PMC3310001; doi:10.1371/journal.pone.0032923)
Supplement: Supporting Information S1 — The detailed methods explaining the use of ancestral genome reconstructions to trace the evolutionary history of individual focal genes. (PDF) [file pone.0032923.s007.pdf]

Supporting Information S1: This file contains the description of how ancestral genome reconstructions were used to reconstruct the duplication history of the RLN/INSL and RXFP gene families.

Using ancestral genome reconstructions to resurrect the duplication history of gene families

Multiple studies have been conducted in the last several years with the goal of understanding the evolution of genomes in the chordate lineage [1]. We used the two most recent ancestral genome reconstruction models by Nakatani *et al.* [2] and Putnam *et al.* [3] (therein referred to as "N" and "P" model respectively, see Figure 1 in main text) to clarify how the three rounds of whole genome duplications (1R, 2R and 3R) and subsequent genome rearrangements could have influenced the evolution of the *RLN/INSL* and *RXFP* families. In addition, we used the work by Kasahara *et al.* [4] to shed light on the effects of teleost-specific genome rearrangements on our genes of interest in medaka, tetraodon and zebrafish. We also referred to the reconstruction of the Eutherian ancestor genome to reconstruct the eutherian state [5]. Because we principally employ the Nakatani *et al.* [2] model, and it includes two alternative scenarios for the rearrangements of some ancestral chromosomes that ensued between the pre-1R to the post 2R vertebrate genomes, in this appendix we also include the alternative scenarios for the gene duplication of our focal genes, which are not shown in main text.

The N-model reconstructs a later stage (compared to the P-model) in the evolution of chordate genome.

Although both the N- and P-models were constructed based on similar methodologies, the models differ in the number of ancestral chromosomes they predict and ultimately reconstruct two different ancestral genomes. In particular, there is a significant difference in the conclusions made by each model about the pre-1R ancestor linkage groups: for example, the number of chordate linkage groups (*CLG*s, P-model) equals 17 while the number of vertebrate ancestral chromosomes (*VAC*s, N-model) is in the range of 10-13. The discrepancies between the two reconstructions can be explained by the inaccuracy of either or both models and by the evolutionary distance between the reconstructed genomes.
Putnam *et al.* [3] compared vertebrate genomes to the genome of amphioxus to reconstruct the linkage groups *ancestral to both amphioxus and vertebrates*, or more accurately, olfactores (ancestor of tunicates and modern vertebrates). On the other hand, Nakatani *et al.* ([2] used protein-coding genes from *Ciona* and sea urchin to *outline* groups of paralogs in vertebrates without directly comparing the synteny between vertebrate and invertebrate genomes.
Overall, it is clear that the P-model reconstructs an earlier stage in the evolution of chordate karyotype (a "pre-1R protokaryotype") compared to the N-model, which shows a pre-1R genome that is structurally very close to its modern vertebrate counterpart. The evolutionary separation between the N- and P-model ("P") genomes should therefore be significant (Figure 1, main text).

Given these assumptions, it can be hypothesized that the amphioxus-olfactores ancestral genome underwent several chromosomal fusions which led to a decrease in the number of chromosomes in the pre-1R vertebrate ancestor from 17 to 10-13 (See below and Figure S3). Alternatively, the difference in the number of linkage groups may be attributable to the inaccuracy of one or both of the models.

How accurate are ancestral reconstructions?

Ancestral reconstructions, like any analyses indeed, are prone to errors. The accuracy of ancestral genome reconstruction is dependent on multiple factors among which the utilized methods and considered evolutionary scales are among the more prominent ones (discussed in [1]). Hence we sought for phylogenetic and small scale synteny data confirmation for all results derived from the tracing of the history of our focal genes in this work.

Tracing of the evolutionary history of genes in vertebrates using the N-model:

First, we mapped all medaka *rln/insl-rxfp* genes to ancestral pre-3R teleost chromosomes (Table S1: a-m). Each of the pre-3R teleost chromosomes as well as the human and chicken chromosomes can be inferred to be composed of *GAC*s (gnathostome ancestor chromosomes, e.g. *A0-A5, J0-J1*), which themselves arose from duplications of the ancestral vertebrate chromosomes *A-J* [2]. This allows one to compare the sets of *GAC*s between human and medaka, and, given that the genomic location of the focal genes are known in human, chicken and the ancestor of medaka, it is then possible to trace the chromosomal origins of the genes in the common ancestor of teleosts, human and chicken (osteichtyan ancestor, see Figure 1 in main text).
Thus, secondly we determined which *GAC*s host each of the *RLN/INSL* and *RXFP* genes. We did this by comparing *GAC*s assigned to each of the genes in the human, medaka and chicken (Table S1: *GAC(H)*, *GAC(M)* and *GAC(C)*) and identifying the ones common to at least 2 of the analyzed genomes. For example, the comparison of the human, medaka and chicken *GAC*s for *RXFP3-1, RXFP3-3* and *RXFP3-4* led us to conclude that these genes originate from 3 post-2R *GAC*s (*A0, A4* and *A5*, respectively) (Table S1). This supported our conclusion about the ohnologous nature of *RXFP3-1*, *RXFP3-3* and *RXFP3-4*, which appear paralogous on the phylogenetic tree (Figure 5, main text).

Genes that exist in only one of the analyzed species were assigned to a *GAC* with the aid of other phylogenetic and syntenic data. For example, the *rxfp3-2* genes, which have been found in all studied teleosts, but have no traceable orthologs in human or chicken, were assigned to *GAC* “A1” using the following rationale. The medaka *rxfp3-2* gene belongs to the pre-3R chromosome “*m*”, which is a mosaic of genes from 7 *GAC*s (*A1, A2, B0, B5, F0, J1* and *E1*) (Table S1). Due to absence of *GAC* data for this gene from human and chicken, it is not possible to deduce the GAC hosting *rxfp3-2* solely based on the information available for medaka. Our phylogeny shows that the teleost *rxfp3-2* genes cluster together, in close proximity, to the *RXFP3-1* cluster (See Figure 5, main text), suggesting that *RXFP3-1* and *3-2* are paralogs. Hence, the next step was to determine whether the teleost *rxfp3-2* gene was ohnologous to vertebrate *RXFP3/4* genes.

Although *RXFP3-2* has no tetrapod orthologs, its neighboring genes do have tetrapod orthologs, and the synteny of these neighboring genes allowed us to estimate the ancestral linkage of *RXFP3-2*. For example, medaka *rxfp3-2a* has two neighboring genes, *sirt6* (sirtuin 6, ENSORLG00000014983) and *eef2* (eukaryotic elongation factor-2, ENSORLG00000015009), and their chicken orthologs (ENSGALG00000001245 and ENSGALG00000001830) are found in chromosome 28 (see ENSEMBL genome browser). Since chicken chromosome 28 is syntenic only to *GAC "A1"* [2], we infer that *RXFP3-2* belongs to *GAC "A1"*. In addition, because the four *RXFP3/4* genes are mapped to 4 duplicated *GAC* chromosomes (*A0, A1, A4* and *A5*), we conclude that they are likely to be ohnologs.

An approach similar to the one described above was used to trace the ancestral origins of *INS/IGF* genes to clarify whether the relaxin and insulin/IGF genes were situated on one pre-1R *VAC* (vertebrate ancestral chromosome) and whether they arose from one ancestral pre-1R gene.

Two scenarios of the duplication and rearrangement history of VAC "A" (N-model)

In their work, Nakatani *et al.* (2007) proposed two scenarios for the duplication and rearrangement history of *VAC "A"*. According to one scenario (the "fission scenario", which we adopt as the framework for our analyses), a single chromosome in the pre-2R vertebrate ancestor is duplicated by 1R to produce two daughter chromosomes. One of these daughter chromosomes is further split into two linkage groups (one of them containing *AncRln-II* and the other- *AncRxfp3-II* in Figure 2, in main text). Hence before the onset of 2R, the post-1R vertebrate genome had a total of 3 *VAC "A"* descendants, which are duplicated by 2R to give rise to six post-2R chromosomes (*GAC "A0-A5"*). According to the alternative scenario of *VAC "A"* evolution (the "fusion scenario", see Figure S1), the pre-2R vertebrate had two chromosomes (*VAC "A-I"* and *VAC "A-II"*), which after 1R yielded four post-1R linkage groups (*A-Ia/b* and *A-IIa/b* in Figure S2). Two of the post-1R chromosomes undergo fusion, which brings the total number of chromosomes down to 3, equaling the number of chromosomes at the onset of 2R described by the first scenario. Identical to the first scenario, 2R yields six *GAC* chromosomes (*GAC "A0-A5"*).

Essentially, the main conclusions (e.g. about the evolutionary relationships among *RLN/INSL* and *RXFP3/4* genes, their WGD-driven origination) of this work are not altered by choosing either of the two scenarios. We adopt the "fission" scenario for our main text because it was chosen by Nakatani *et al.* [2] for the figure in their manuscript depicting the reconstructed genome of the pre-2R vertebrate ancestor using the least possible number (10 as opposed to 13) of chromosomes.
The important difference between the two scenarios is the ancestral linkage of the *AncRln-like* ligand and *AncRxfp3/4* receptor genes (compare Figures 2 and S1).

Our conclusions (N-model):

- Good-Avila *et al.* [1] previously demonstrated that the *RLN/INSL* genes of teleosts and human are orthologous. Here we confirmed the synteny among the human, medaka

and chicken genes (along with other vertebrate genes, see Appendix B), and by mapping them to the N-model we show that *RLN(2), RLN3, INSL3* and *INSL5* originated from one gene, which we call *AncRln-like*, in the pre-1R vertebrate ancestor and that they multiplied into four loci commensurate with the 2R events. Thus these 4 loci can be described as "ohnologs" based on their WGD-related evolutionary descent. All four *RLN/INSL* genes arose as a result of 2R. After 1R, the *AncRln-like* gene duplicated giving rise, in the first instance, to the ancestor of the *RLN/INSL3* genes and, in the second instance, to the ancestor of the *RLN3/INSL5* genes. After 2R, these ancestral genes again duplicated giving rise to the 4 genes common to teleosts and tetrapods: *RLN, INSL3, RLN3* and *INSL5* (Figure 1, main text).

- RXFP3 and RXFP4 receptors arose from one ancestral gene. All *RXFP3/4* genes are 2R-ohnologs.

• Both *RLN/INSL* and *RXFP3/RXFP4* genes originated from one *VAC* named "A" by Nakatani *et al.* [2]. While *RLN(2)* and *INSL3* can be traced to the same gnathostome ancestor chromosomes (*GAC*s) as *RXFP3-1* and *RXFP3-2, RLN3, INSL5, RXFP3-3* and *RXFP4* are situated on different *GAC*s. According to the fission scenario, a logical explanation for this is that the pre-1R vertebrate ancestor had one *RLN3/INSL5*-like gene and one *RXFP3/RXFP4*-like gene which were linked on one chromosome. 2R duplicated the genes, but chromosomal rearrangements disrupted their linkage, thus the ligands and receptors were unlinked at the end of 2R.

• *RXFP1* and *RXFP2* are ohnologs.

• *RXFP1/2* and *RXFP2-like* originated from 2 *VAC*s that are different from that hosting *RXFP3/RXFP4* and *RLN/INSL* genes (*VAC "A"*). These chromosomes are known as "C" (*AncRxfp1/2*) and B or F (*AncRxfp2-like*). See main text for the discussion of the *Rxfp2-like* origins.

• Two different scenarios could explain the origin of *RXFP1/RXFP2*: these are shown in Figure S2.

• Although the tracing of the *INS/IGF* genes was problematic due to insufficient data available for medaka and other teleosts, these genes seem to have originated from an ancestral vertebrate chromosome *"D"* that is different from both *VAC "A"* and *"C"* that carried the ancestors of *RLN/INSL* and *RXFP* genes.

Search for the evidence of the presence of regions orthologous to *RLN/INSL* and *RXFP* loci in the amphioxus ancestor using the P-model:

Using their known genomic locations, each of the human *RLN/INSL* and *RXFP* genes were mapped to a chromosomal segment (Table S3: "Segment ID"). The identified chromosomal segments were then traced to *CLG*s using the oxford grid provided [3]. Additionally, the scaffold locations of amphioxus *ilp* and *rxfp1/2*-type genes were also traced, where possible, to *CLG*s using the oxford grid (Table S3). Since the oxford grid incorporates map locations from only two organisms, i.e. human and amphioxus, and because the identities of the amphioxus genes are still to be established, this method allowed us to use the genomic information pertaining only to the genes present in the human genome. In other words, the *CLG* origins of genes such as *Rxfp3-2* that have not been identified in humans (but exist in teleosts, for example) could not be traced using this model.

Our conclusions (P-model):

- All human *RLN/INSL* genes were traced to the same chordate linkage group (*CLG*), *CLG1,* agreeing with the N-model that all RLN family genes arose from a single ancestral gene.
- Only *RXFP3-1* was traced to *CLG1*, *RXFP3-3* was localized to *CLG2*, and the location of *RXFP4* is unclear.
- Both *RXFP1* and *RXFP2* were mapped to *CLG8*, while *RXFP2-like* was mapped to *CLG*9
- *INS* and *IGF2* were clearly mapped to a *CLG* different from those occupied by the *RLN/RXFP* genes confirming that the ancestral *INS/IGF2* and *RLN/INSL* have separate ancestral chromosome origins. Also one could conclude that the ancestral *INS/IGF2* genes were in a separate linkage group from ancestral *RLN/INSL* before the split of the amphioxus and olfactores lineages. (Following from this conclusion it is tempting to revisit the identities of the three *INS/IGF/RLN*-like genes previously identified in *C. intestinalis* as linked on one chromosome [6]).
- Some of the amphioxus candidate *rln/insl*, *ins/igf* and *rxfp1/2* genes that we obtained from public databases (Table S1 and S10) were assigned to the same *CLG*s as their human counterparts (the *ins/igf-like* and *rxfp1/2-like* groups). We were unable to identify any *rxfp3/4-like* genes in the amphioxus databases.

Gene gain/loss and genomic rearrangements in the pre-2R ancestor and/or inaccuracy of ancestral genome reconstruction models may account for the difference in the results obtained using the two models:

According to the results of the gene tracing method using the P-model, *RXFP3* and *RXFP4*-type genes originate from at least 2 different *CLG*s and only one of them, *RXFP3-1*, appears to have been linked to the ancestral *RLN/INSL* gene on *CLG1*. This would suggest that *RXFP4* has a different evolutionary origin from *RXFP3*. On the contrary, the N-model gene tracing method predicts that all *RXFP4* and *RXFP3*-type genes originated from one ancestral receptor gene that was linked to the ancestral *RLN/INSL* gene (*VAC "A"*, as described above).
How can this disagreement be explained?
As discussed above, the ancestor linkage groups reconstructed in the P- and N-models are not equivalent. It is possible that some of the *CLG*s of the amphioxus-olfactores ancestor fused to produce "multi-*CLG*" chromosomes of the vertebrate ancestor. For instance, *CLG1*, *CLG2* and could have fused together and with other unknown *CLG*s, resulting in the so-called *VAC "A"* reconstructed by Nakatani *et al.* [2] Intriguingly, amphioxus does not seem to possess *rxfp3/4*-type genes which implies that these genes appeared after the divergence of cephalochordates.
Alternatively the observed discrepancy could stem from inaccurate ancestral genome reconstruction.

References

1. Muffato M, Roest Crollius H (2008) Paleogenomics in vertebrates, or the recovery of lost genomes from the mist of time. BioEssays 30: 122–134.

2. Nakatani Y, Takeda H, Kohara Y, Morishita S (2007) Reconstruction of the vertebrate ancestral genome reveals dynamic genome reorganization in early vertebrates. Genome Res 17: 1254–1265.

3. Putnam NH, Butts T, Ferrier DEK, Furlong RF, Hellsten U, et al. (2008) The amphioxus genome and the evolution of the chordate karyotype. Nature 453: 1064–1071.

4. Kasahara M, Naruse K, Sasaki S, Nakatani Y, Qu W, et al. (2007) The medaka draft genome and insights into vertebrate genome evolution. Nature 447: 714–719.

5. Kemkemer C, Kohn M, Cooper DN, Froenicke L, Högel J, et al. (2009) Gene synteny comparisons between different vertebrates provide new insights into breakage and fusion events during mammalian karyotype evolution. BMC Evol Biol 9: 84.

6. Olinski RP, Lundin L-G, Hallböök F (2006) Conserved synteny between the Ciona genome and human paralogons identifies large duplication events in the molecular evolution of the insulin-relaxin gene family. Mol Biol Evol 23: 10–22.
