# Supplementary material for: Using Paleogenomics to Study the Evolution of Gene Families: Origin and Duplication History of the Relaxin Family Hormones and Their Receptors
Source: PLoS One. 2012 Mar 21;7(3):e32923. doi: 10.1371/journal.pone.0032923 (PMC3310001; doi:10.1371/journal.pone.0032923)
Supplement: Dataset S1 — All accession numbers and map locations for genes used in this study (includes Tables S1–S12). (PDF) [file pone.0032923.s008.pdf]

Table S1. Dataset used to reconstruct the duplication history of *RLN/INSL-RXFP* genes using model "N"

Map positions of *RLN/INSL*, *RXFP* and *INS/IGF* genes in human (H), medaka (M) and chicken (C) are shown. The pre-3R teleost ancestor chromosomes that gave rise to the *rln/rxfp* medaka chromosomes are shown in column 3 (pre3R)
CVLb (H): Conserved Vertebrate Linkage blocks in human, determined using the known intrachromosomal position of human genes and the protochromosome map scheme from Nakatani et al. (2007)
GAC: Gnathostome Ancestor Chromosomes, reconstructed by Nakatani et al. (2007)
GAC (H): GACs potentially hosting the genes of interest based on the identified CVLb (H)
GAC (C): GACs hosting *RLN/INSL-RXFP*genes in chicken
GAC (R): GAC Resolved to contain *RLN/INSL-RXFP* genes in the gnathostome ancestor
GAC (M): GACs hosting the genes of interest based on the comparison between medaka and human
CVLb (R): CVLb resolved to contain *Rln/Insl-Rxfp* genes in the gnathostome ancestor

	Map positions				Ancestral linkage groups						
**Gene**	**H**	**C**	**M***	**pre-3R**	**CVLb (H)****	**GAC (H)**	**GAC (M)**	**GAC (C)**	**CVLb (R)****	**GAC (R)**	**VAC**
RLN (2)	9: 5.30	Z: 28.2	12: 14.80	*i*	50	*A0*	*A0*	*A0*	50	*A0*	***A***
INSL3	19: 17.93	n/a	17: 60.74	*m*	89-90	*A3-A1*	*A1*	n/a	90	*A1*	
INSL5	1: 67.26	8: 29.36	4: 16.56; 17: 16.79	*m*	2	*A2*	*A2*	*A2*	2	*A2*	
RLN3	19: 14.14	n/a	1: 31.36; 8: 30.65	*f-e-d*	89	*A3*	*A3 (e)*	n/a	89	*A3*	
RXFP3-1	5: 33.93	Z: 9.7	12: 10.00	*i*	27	*A0*	*A0*	*A0*	27	*A0*	***A***
RXFP3-2	n/a	n/a	17: 22.92; 4: 8.24	*m; m*	n/a	n/a	*A1-A2-B0-B5-F0-J1-E1*	n/a	88-90-111	*A1*	
RXFP3-3	15: 79.12†	n/a	3: 10.20-10.95; 6:?	*j; j-k*	76	*A4*	*A4 (j)*	n/a	76	*A4*	
RXFP4	1: 155.91	n/a	11: 6.39	*b*	4-5-109	*A5-A2-Un*	*A5*	n/a	4	*A5*	
RXFP1	4: 159.24	4: 23.0	10: ?	*f-g*	25	*C1*	*C1*	*C1*	25	*C1*	***C***
RXFP2	13: 32.31	1: 179.1†	14: 9.82	*g-h*	71	*C0*	*C0*	*C0*	71	*C0*	
RXFP2-like	X: 67.94†	4: 0.57	n/a	n/a	108-117	*B0-F4*	n/a	*C1-B0-F4*	108-117	*B0-F4*	***B/F***

Table S2. Dataset used to reconstruct the duplication history of *Ins/Igf* genes using model "N"

Tetrapods: Human and Chicken

						Ancestral linkage groups	
**Gene**	**H**	**C**	**CVLb (H)**	**GAC (H)**	**GAC (C)**	**CVLb (R)**	**GAC (R)**
INS1	11: 2.153	5: 14.85	60	D1	D1	60	*D1*
IGF2	11: 2.150	5: 14.88	60	D1	D1	60	*D1*
IGF1	12: 102.79	1: 57.33	68	D0	D0	68	*D0*

Fish: Medaka (M) and Tetraodon (T)

	**T**	**pre-3R (T)**	**M^**	**pre-3R (M)**	**pre-3R M+T*****	**GAC (R)**
ins1	1: 3.38	*f/g/m*	21: ?	*c?*	*g*	**B0/C2/F4**
ins2	7: 10.6	*g/h*	14: ?	*g-h?*	*g*	**B0/C2/F4**
igf1	19:1.39	*k*	23: 20.44	*k*	*k*	**D0**
igf2	13: 6.6	*j/k*	n/a	*?*	*k*	**D0**

* two positions are given for 3R duplicates located on two separate modern medaka chromosomes originating from one pre3R teleost ancestor chromosome
** when multiple blocks match the same location/GAC, all possible block numbers are given separated by dashes (e.g. #-#-#)
† pseudogene
^ not all genome regions hosting Ins/Igf genes in medaka are assembled into chromosomes, therefore mapping of these genes is imprecise
*** based on shared ancestral teleost linkage groups, since all Tetraodon genes can be mapped to distinct chromosomes, results obtained for Tetraodon overrode Medaka
Genomic region around Tetraodon Ins1 is syntenic to the region of Human Chromosome 5 under CVLb 28, which is equivalent to C2

Table S3. Dataset used to reconstruct the origins of *Rln/Insl-Rxfp* genes in the chordate ancestor using model "P"

Human genes were mapped to genome segments determined by Putnam *et al*., amphioxus candidate gene locations (scaffolds) were also assigned to CLGs based on the Oxford grid in Putnam *et al*.
Segment ID: Human Genome Segment boundaries corresponding to genomic locations as described in Putnam *et al.* [2]
CLG: Chordate Linkage Group, Putnam *et al.* identified 17 CLGs which correspond to the hypothetical chromosomes of the ancestor of amphioxus and *Olfactores* (tunicates and vertebrates)

Human genes

**Gene**	**Location**	**Segment ID**	**CLG**
RLN (2)	9: 5.30	9.1	1
INSL3	19: 17.93	19.3	1
INSL5	1: 67.26	1.5	1
RLN3	19: 14.14	19.2	1
RXFP3-1	5: 33.93	5.1	1
RXFP3-3	15: 79.12†	15.2	2
RXFP4	1: 155.91	1.13	un*
RXFP1	4: 159.24	4.4	8
RXFP2	13: 32.31	13.1	8
RXFP2-lik	X: 67.94†	X.5	9
INS	11: 2.153	11.1	14
IGF2	11: 2.150	11.1	14
IGF1	12: 102.79	12.9	un*

Amphioxus rln/insl/rxfp and ins/igf candidates

	**Gene ID**	**Scaffold**	**CLG**
ilp4	7253642	bf_v2_277	un*
ilp5	7255900	bf_v2_277	un*
ilp6	7230317	bf_v2_196	4
ilp2	***n/a***	bf_V2_190	14
ilp1	7235917	bf_V2_190	14
ilp3	7251652	bf_v2_243	14
rxfp1/2-like	7252026	bf_v2_249	7-8
	7229038	bf_v2_150	8
	7221608	bf_v2_150	8
	7207790	bf_v2_21	un*
	7209355	bf_v2_21	un*

*segment not mapped to a CLG

Table S4. Relaxin family peptide/receptor genes and their IDs in tetrapods (20 species)

Chromosomal locations given in [chromosome number: megabases (Mb), rounded to the nearest 100.000 bp], Ensembl or GenBank IDs given.

Legend (colour key):
- gene name needs to be updated on ensembl
- not found or a pseudogene
- found but not annotated
- NCBI

					RLN-locus							
	RLN3	*INSL3*	*INSL5*	*RLN*[a]	*INSL4*	*INSL6*	*RXFP1*	*RXFP2*	*RXFP2-like*	*RXFP3-1*[n]	*RXFP3-3*	*RXFP4*
**Placental mammals**												
1 *H. sapiens (human)*	ENSG00000171136	ENSG00000248099	ENSG00000172410	ENSG00000107014	ENSG00000120211	ENSG00000120210	ENSG00000171509	ENSG00000133105	†	ENSG00000182631	GENSCAN00000038299^	ENSG00000173080
2 *M. mulatta (rhesus)*	ID: 717577	HM102325	ID: 699803	ID: 693473	ID: 693911	ID: 693735	ID: 701107	ID: 721969	†?	ID: 698115	ID: 100426387†	ID: 718025
3 *B. taurus (cow)*	ENSBTAG00000038437	ENSBTAG00000025775	ENSBTAG00000003850	n/a	n/a	ENSBTAG00000006651	ENSBTAG00000010306	ENSBTAG00000015132	†?	ENSBTAG00000039929	ENSBTAG00000026976	ID: 450212
4 *S. scrofa (pig)*	ENSSSCG00000013765	ENSSSCG00000013887	n/a?	ENSSSCG00000005216/	n/a	ENSSSCG00000005214	ENSSSCG00000008875	ENSSSCG00000009336	†?	ENSSSCG00000016820	ENSSSCG00000001768	ENSSSCG00000006503
5 *E. caballus (horse)*	ENSECAG00000014897	ENSECAG00000016450	ENSECAG00000024174	ENSECAG00000013020	n/a	ID: G7100146379	ENSECAG00000013594	ENSECAG00000014103	†?	ENSECAG00000012797	†?	ENSECAG00000007292
6 *C. lupus (dog)*	ID: 610834	ENSCAFG00000015187	†	ENSCAFG00000002115	n/a	ENSCAFG00000002113	ENSCAFG00000008672	ENSCAFG00000006501	†?	ID: 489237	†?	†
7 *S. araneus (shrew)*	ENSSARG00000011917	****	†	ENSSART00000013075	n/a	ENSSARG00000004569	ENSSARG00000013665	ENSSARG00000009883	†?	ENSSARG00000000381	†?	n/a
8 *C. porcellus (guinea pig)*	ENSCPOG00000013564	ENSCPOG00000019362	ENSCPOG00000011735	ENSCPOG00000001365	n/a	ENSCPOG00000002694	ENSCPOG00000015517	ENSCPOG00000009157	†?	ENSCPOG00000009428	†?	ENSCPOG00000003454
9 *M. musculus (mouse)*	ENSMUSG00000045232	ENSMUSG00000079019	ENSMUSG00000066090	ENSMUSG00000039097	n/a	ENSMUSG00000050957	ENSMUSG00000034009	ENSMUSG00000053368	†?	ENSMUSG00000060735	†?	ENSMUSG00000049741
10 *R. rattus (rat)*	ENSRNOG00000005911	ENSRNOG00000018757	ENSRNOG00000037916†	ENSRNOG00000015920	n/a	ENSRNOG00000015868	ENSRNOG00000024120	ENSRNOG00000000897	†?	ENSRNOG00000023126	†?	†
11 *O. cuniculus (rabbit)*	ENSOCUG00000013451	n/a?	ID:100141505	ENSOCUG00000027403	n/a	ENSOCUG00000013682	ENSOCUG00000001208	ENSOCUG00000000751	†?	ENSOCUG00000004946	†?	ENSOCUG00000026819
12 *D. novemcinctus (armadillo)*	ENSDNOG00000012940	no ID	ENSDNOG00000015104	ENSDNOG00000025594	n/a	ENSDNOG00000001668	ENSDNOG00000001016	ENSDNOG00000018859	†?	ENSDNOG00000012031	†?	ENSDNOG00000017055
13 *L. africana (elephant)*	no id	ENSLAFG00000025675	ENSLAFG00000015826	ENSLAFG00000017411	n/a	ENSLAFG00000007479	ENSLAFG00000014435	ENSLAFG00000012550	†?	ENSLAFG00000017082	†?	ENSLAFG00000011205
**Marsupial mammals**												
14 *M. domestica (opossum)*	gi|126323337	gi|126323992	no id	ENSMODG00000015357	n/a	n/a	ENSMODG00000001973	ENSMODG00000009382	ENSMODG00000012676	ENSMODG00000020402	ENSMODG00000025367	ENSMODG00000024291
**Monotreme mammals**												
15 *O. anatynus (platypus)*	gi|170014739	ENSOANG00000021585	n/a	gi|170014735	n/a	n/a	ENSOANG00000005387	ENSOANG00000015476		ENSOANG00000001969	†?	n/a
**Reptiles**												
16 *A. carolinensis (lizard)*	ENSACAG00000015658	n/a	ENSACAG00000011316	GENSCAN00000002500	n/a	n/a	ENSACAG00000016552	n/a	ENSACAG00000007727	ENSACAG00000008321	n/a	n/a
**Birds**												
17 *G. gallus (chicken)*	n/a	n/a	ENSGALG00000020599	ENSGALG00000015028	n/a	n/a	ENSGALG00000009429	n/a	ENSGALG00000004543	ENSGALG00000017411	n/a	n/a
18 *T. guttata (zebrafinch)*	n/a	n/a	ENSTGUG00000010160	ENSTGUG00000005041	n/a	n/a	ENSTGUG00000005573	n/a	n/a	ENSTGUG00000001946	n/a	n/a
**Amphibia**												
19 *X. laevis (frog)*	EU437449	ENSXETG00000016437	no id	ENSXETG00000000587	n/a	n/a	ENSXETG00000019493	ENSXETG00000019186	ENSXETG00000011511	ENSXETG00000011511	n/a	ENSXETG00000001632
20 *R. esulenta (edible frog)*◊	FJ230963.2	AJ298874.1										

[n] **in** humans RXFP3-1 and RXFP3-4 are equivalent to functional RXFP3 and RXFP4 respectively
**identical one found at 1:226.8
†: putative pseudogene
***predicted by Genscan
◊ annotated in NCBI

n/a: not identified and gene probably does not exist.
n/a? not identified because region not well annotated/sequenced, but sequence may exist
: RLN is misnamed as RLN3 on Ensembl/NCBI, synteny confirms orthology to mammalian RLN
¥ Rxfp2 gene is split between 2 locations: the LDL module is on chr12 and the LRR/7tm is on the scaffold
^ genescan prediction incorrect but includes part of gene
****there, but contig not fully sequenced and cannot get sequence

Table S5. Mammalian lineage-specific duplicates in the *RLN*-locus

**Human**	**ID**
RLN1	ENSG00000107018
RLN2	ENSG00000107014
**Rabbit**	
Relaxin-like protein SQ10 Precursor	ENSOCUG00000027934
Relaxin-like protein SQ10 Precursor	ENSOCUG00000027403
unnamed	ENSOCUG00000026316
unnamed	ENSOCUG00000008099
unnamed	ENSOCUG00000008103
unnamed	ENSOCUG00000025640
**Shrew**	
unnamed	ENSSARG00000013075
unnamed	ENSSARG00000004245
unnamed	ENSSARG00000010511
**Armadillo**	
rln-a	ENSDNOG00000025594
unnamed	ENSDNOG00000025720
**Pig**	
Prorelaxin Precursor	ENSSSCG00000005216
Relaxin R-II1 A chain	ENSSSCG00000005213

Table S6. Relaxin family peptide/receptor genes and their chromosomal locations in tetrapods (20 species)

Chromosomal locations given in [chromosome number: megabases (Mb), rounded to the nearest 100.000 bp], all locations are from Ensembl, unless otherwise specified

					RLN-locus								
		RLN3	***INSL3***	***INSL5***	***RLN***	***INSL4***	***INSL6***	***RXFP1***	***RXFP2***	***RXFP2-like***	***RXFP3-1ⁿ***	***RXFP3-3***	***RXFP3-4ⁿ***
	**Placental mammals**												
1	*H. sapiens (human)*	19: 14.14	19: 17.93	1: 67.26	9: 5.30	9: 5.23	9: 5.16	4: 159.24	13: 32.31	X: 67.94†	5: 33.93	15: 79.12†	1: 155.91
2	*M. mulatta (rhesus)*	19: 13.71	19: 17.4	1: 69.58	15: 71.83	15: 71.92	15: 72.00	5: 15.07	17: 11.10	†?	6: 34.04	7: 57.95†	1: 13.45
3	*B. taurus (cow)*	7: 9.87	7: 5.26	3: 84.21	n/a	n/a	8: 41.52	17: 42.34	12: 29.01	†?	20: 42.31	21: 30.35	3: 16.15
4	*S. scrofa (pig)*	2:57.0	2:62.1	6*	1:226.8**	n/a	1:226.74	8:41.1	11:7.7	†?	16:17.5	7: 54.0	4: 98.0
5	*E. caballus (horse)*	7:44.8	21:2.6	5:94.1	23:26.7	n/a	23:26.6	2:75.8	17:11.0	†?	21:30.7	1: 115.6†	5: 42.2
6	*C. lupus (dog)*	20:51.5***	20:48.1	5:46.7†	1: 96.6	n/a	1: 96.5	15: 58.7	25: 11.3	†?	4: 77.1◊	†?	7: 44.8†
7	*S. araneus (shrew)*	sc79318: 0.0006:	sc_1461:1032	†?	scaffold_70875: 4	n/a	sc2354: 0.012	sc5672: 0.004	sc3003: 0.1	†?	sc4216: 0.008	†?	n/a?
8	*C. porcellus (guinea pig)*	sc42: 12.8	sc72: 7.8	sc2: 66.3	sc21: 14.7	n/a	sc21: 14.7	sc7: 31.8	sc6: 33.2	†?	sc29:21.7	†?	10:16.6
9	*M. musculus (mouse)*	8: 86.6	8: 74.2	4: 102.7	19: 29.4	n/a	19:29.4	3: 79.5	5: 150.8	†?	15: 10.96	†?	3: 88.5
10	*R. rattus (rat)*	19: 25.8	16: 18.9	5: 123.9†	1: 233.0	n/a	1: 233.0	2: 171.1	12: 5.3	†?	2: 60.4	†?	2: 180.8†
11	*O. cuniculus (rabbit)*	sc1049: 0.028	n/a?	13:99.4	chr1	n/a	chr1	sc58: 1.2	sc59: 2.4	†?	11: 55.7	†?	13:36.9
12	*D. novemcinctus (armadillo)*	sc236409: 0.0001	sc31772: 0.017	sc2247: 0.3	sc1575: 0.061	n/a	sc119039: 0.003	sc1259: 0.003	sc3001: 0.024	†?	sc6302:0.04†	†?	sc4957:0.006
13	*L. africana (elephant)*	sc26: 29.1	sc26: 24.2	sc17: 27.1	sc6: 93.9	n/a	sc88: 0.58	sc61: 9.6	sc11: 5.3	†?	sc7: 37.9	†?	sc33: 4.4
	**Marsupial mammals**												
14	*M. domestica (opossum)*	3: 446.3	3: 476.4	2: 24.49	6: 166.9	n/a	n/a	5: 116.5	4: 303.2	Un: 50.4, X?	3: 242.6	1: 9.8	2: 190.8
	**Monotreme mammals**												
15	*O. anatynus (platypus)*	ultc605:0.02	c19353: 0.007	n/a?	X5: 11.5	n/a	n/a	12: 13.5/c5907:	ultc336: 0.46¥	?	c1755: 0.011	†?	n/a?
	**Reptiles**												
16	*A. carolinensis (lizard)*	sc132: 2.7	n/a?	sc619: 0.48	sc13: 3.2	n/a	n/a	sc284: 0.6	n/a	sc1398: 0.05	sc3: 1.7	n/a	n/a?
	**Birds**												
17	*G. gallus (chicken)*	n/a?	n/a?	8: 29.36	Z: 28.2	n/a	n/a	4: 23.0	1: 179,14 †	4: 0.57	Z: 9.7	n/a	n/a
18	*T. guttata (zebrafinch)*	n/a?	n/a?	8: 26.7	Z: 63.9	n/a	n/a	4: 29.7	n/a	n/a?	Z: 41.0	n/a	n/a
	**Amphibia**												
19	*X. laevis (frog)*	sc649: 0.087	sc969: 0.20	sc431: 0.98	sc86: 2.1	n/a	n/a	sc110: 0.6	sc80: 1.6	sc422: 0.30	sc803: 0.128	n/a	sc9769: 0.009
20	*R. esulenta (edible frog)◊*	FJ230963.2	AJ298874.1										

ⁿ **in** humans RXFP3-1 and RXFP3-4 are equivalent to functional RXFP3 and RXFP4 respectively

* not annotated along with syntenic genes, but probably exists

**identical one found at 1:226.8

†: putative pseudogene: rat insl5 is a processed pseudogene

***predicted by Genscan

◊ annotated on NCBI

n/a?: not identified, but likely exists

: RLN is misnamed as RLN3 on Ensembl/NCBI, synteny confirms orthology to mammalian RLN

¥ Rxfp2 gene is split between 2 locations: the LDL module is on chr12 and the LRR/7tm is on the scaffold

Table S7. Relaxin family peptide/receptor genes and their IDs in teleost fish (5 species)

Chromosomal locations given in [chromosome number: megabases (Mb), rounded to the nearest 100.000 bp], all locations are from Ensembl, unless otherwise specified

rln/insl genes

Species	*rln3a*	*rln3b*	*insl3*	*insl5a*	*insl5b*	*rln*[a]
D. rerio (zebrafish)	ENSDARG00000070780	ENSDARG00000039854	ENSDARG00000035862	ENSDARG00000070966	ENSDARG00000069294	100329416
O. latipes (medaka)	ENSORLG00000011777	ENSORLG00000010278	GENSCAN00000085130	GENSCAN00000098652	NO ID	ENSORLG00000009974
G. aculeatus (stickleback)	ENSGACG00000018985	ENSGACG00000012435	NO ID	NO ID	ENSGACG00000016154	ENSGACG00000017364
T. nigroviridis (tetraodon)	GSTENG00026277001	†?	GSTENG00020897001	EU437461	EU437463	EU437459
T. rubripes (takifugu)	ENSTRUG00000010031	ENSTRUG00000012677	SINFRUG00000162280	GENSCAN00000013221	GENSCAN00000015952	ENSTRUG00000005640

rxfp1/2 and rxfp3/4 genes

Species	*rxfp1*	*rxfp2a*	*rxfp2b*	*rxfp2-like*	*rxfp3-1*	*rxfp3-2a*
D. rerio (zebrafish)	ENSDARG00000090071	ENSDARG00000032820	ENSDARG00000019660	ENSDARG00000068731	ENSDARG00000057410	**ENSDARG00000022739**
O. latipes (medaka)			n/a	n/a	ENSORLG00000006539	ENSORLG00000014985
G. aculeatus (stickleback)	ENSGACG00000016581	ENSGACG00000020550	n/a	n/a	ENSGACG00000016296	ENSGACG00000015315
T. nigroviridis (tetraodon)	ENSTNIG00000013038	ENSTNIG00000009913	n/a	n/a	ENSTNIT00000003086	ENSTNIG00000015329
T. rubripes (takifugu)	ENSTRUG00000016132	ENSTRUT00000005652	n/a	n/a	ENSTRUG00000014489	ENSTRUG00000007126

Species	*rxfp3-2b*	*rxfp3-3a2*	*rxfp3-3a1*	*rxfp3-3b*	*rxfp3-3a3*	*rxfp4*
D. rerio (zebrafish)	ENSDARG00000061846	ENSDARG00000062111	ENSDARG00000069028	ENSDARG00000059348	ENSDARG00000069246	
O. latipes (medaka)	no id	ENSORLG00000001754	ENSORLG00000002054	ENSORLG00000019204	n/a	ENSORLG00000003213
G. aculeatus (stickleback)	ENSGACG00000012856	ENSGACG00000016895	ENSGACG00000016952	ENSGACG00000008049	n/a	ENSGACG00000003931
T. nigroviridis (tetraodon)	ENSTNIG00000010632	ENSTNIG00000009550	ENSTNIG00000009561			ENSTNIG00000009161
T. rubripes (takifugu)	ENSTRUG00000017932	ENSTRUG00000016840	ENSTRUG00000016739	ENSTRUG00000014434	n/a	gi|74096006

Table S8. Relaxin family peptide/receptor genes and their chromosomal locations in teleost fish

Chromosomal locations given in [chromosome number: megabases (Mb), rounded to the nearest 100.000 bp], all locations are from Ensembl, unless otherwise specified

Species	*rln3a*	*rln3b*	*insl3*	*insl5a*	*insl5b*	*rln*[a]
D. rerio (zebrafish)	3: 19.03	1: 50.17	2: 20.05	6: 30.40	2: 9.92	21: 0.20
O. latipes (medaka)	1: 31.36	8: 30.65	17: 60.74	4: 16.56	17: 16.79	12: 14.80
G. aculeatus (stickleback)	9: 15.05	11: 11.63	3: 8.50	8: 8.10	3: 9.58	14: 7.44
T. nigroviridis (tetraodon)	18: 2.04	n/a	15: 3.88	1: 15.66	15: 3.44	4: 1.08
T. rubripes (takifugu)	sc189: 0.355	sc141: 0.678	sc212: 0.364	sc55: 0.131	sc166: 0.545	sc243: 0.093

Species	*rxfp1*	*rxfp2a*	*rxfp2b*	*rxfp2-like*	*rxfp3-1*	*rxfp3-2a*
D. rerio (zebrafish)	14: 51.06	10: 34.81	15: 30.98	5: 37.50	21: 18.31	2: 53.78 (ENSEM), 4:? (NCBI)
O. latipes (medaka)	10: ?*	14: 9.82	n/a	n/a	12: 10.00	17: 22.92
G. aculeatus (stickleback)	4: 2.39	7: 19.90	n/a	n/a	14: 3.51	3: 7.51
T. nigroviridis (tetraodon)	20: 2.24	7: 3.13	n/a	n/a	4: ?†	15: 4.70
T. rubripes (takifugu)	sc5: 0.003	sc2436: 0.007	n/a	n/a	sc44: 1.50	sc200: 0.130

Species	*rxfp3-2b*	*rxfp3-3a2*	*rxfp3-3a1*	*rxfp3-3b*	*rxfp3-3a3*	*rxfp4*
D. rerio (zebrafish)	22: 20.42	7: 58.58	7: 35.21	25: 1.41	18: 19.81	n/a
O. latipes (medaka)	4: 8.24	3: 10.95	3: 10.20	6: ?	n/a	11: 6.39
G. aculeatus (stickleback)	8: 16.28	2: 18.72	2: 19.11	19: 9.18	n/a	10: 5.35
T. nigroviridis (tetraodon)	1: 13.78	5: 10.38	5: 10.62	13: 12.75	n/a	21: 1.73
T. rubripes (takifugu)	sc25: 0.761	sc1: 3.39	sc1: 3.12	sc4528: 0.004	n/a	sc138: 0.096◊

[a] rln is the orthologue of the human RLN2 (H2) gene

†: putative pseudogene

◊ annotated on NCBI as GPR100

* ultracontig115: 1,259,584-1,305,610 (Ensembl) corresponds to chr10 on UTGB

Table S9. Relaxin family peptide/receptor genes and their chromosomal locations in Ascidians (1 species- C.intestinalis)

	Species	Name on phylogeny	Gene ID (Ensembl)	Gene ID (NCBI)	Map location
rxfp1/2-like*	*C. intestinalis*	*rxfp1/2-L*	n/a	100178659	3p: 3.28
rxfp3-4-like	*C. intestinalis*	*rxfp3-L1*	ENSCING00000012511	?	1q: 4.67
		rxfp3-L2	ENSCING00000014875	100185531	6q: 0.975

* rxfp1/2 gene obtained from Kamesh et al. and verified using the Ensembl C. intestinalis browser

Table S10. *Rxfp1/2*-type genes and their chromosomal locations in lancelet (1 species)

Branchiostoma floridae (genome sequenced and assembled into contigs)

	Name on phyloger	Gene ID (NCBI)	Protein ID (JGI)	Scaffold: version2		
rxfp1/2-type* **	*rxfp1/2-L1*	7252026	63628	bf_v2_249		
	rxfp1/2-L2	7229038	134702	bf_v2_150		
	rxfp1/2-L3	7221608	63627	bf_v2_150		
	rxfp1/2-L4	7207790	204729	bf_v2_21		
	rxfp1/2-L5	7209355	98820	bf_v2_21		
		**Gene ID (NCBI)**	**Protein ID (JGI)**	**Scaffold: version2**	**Scaffold: version1***	**Exact map location**
ilps	n/a	n/a**		bf_V2_190	59	
	n/a	7235917	121099	bf_V2_190	59	scaffold_59:2166507-2182058
	n/a	7251652	97394	bf_v2_243	302 (243)	scaffold_302:862279-904829
	n/a	7253642	100967	bf_v2_277	41 (372)	
	n/a	7255900	100968	bf_v2_277	41 (372)	scaffold_372:110792-121518
	n/a	7230317	74371	bf_v2_196	73 (50)	scaffold_50:2135877-2137051

***rxfp1/2 sequences were obtained from NCBI referring to Nordström *et al.*, with the exception of BRAFLDRAFT_231009, which could not be found in the current database

bf_v2_21 shares 2 genes with rxfp2 region

Table S11. *Rxfp1/2*-type genes in Echinoderms (1 species)

Sea Urchin (Strongylocentrotus purpuratus) rxfp1/2 IDs are from SpBase (http://www.spbase.org/SpBase/)

	Species	Name on phylogeny	Gene ID
rxfp1/2s	*S. purpuratus (sea urchin)*	rxfp1/2-L1	SPU_000792
		rxfp1/2-L2	SPU_000840
		rxfp1/2-L3	SPU_001502
		rxfp1/2-L4	SPU_003492
		rxfp1/2-L5	SPU_003527
		rxfp1/2-L6	SPU_004308
		rxfp1/2-L7	SPU_005497
		rxfp1/2-L8	SPU_009179
		rxfp1/2-L9	SPU_011953
		rxfp1/2-L10	SPU_013866
		rxfp1/2-L11	SPU_015134
		rxfp1/2-L12	SPU_016206
		rxfp1/2-L13	SPU_019187
		rxfp1/2-L14	SPU_019188
		rxfp1/2-L15	SPU_019240
		rxfp1/2-L16	SPU_019676
		rxfp1/2-L17	SPU_020381
		rxfp1/2-L18	SPU_020408
		rxfp1/2-L19	SPU_022337
		rxfp1/2-L20	SPU_023168
		rxfp1/2-L21	SPU_023629
		rxfp1/2-L22	SPU_024157
		rxfp1/2-L23	SPU_025906
		rxfp1/2-L24	SPU_026409
		rxfp1/2-L25	SPU_027093
		rxfp1/2-L26	SPU_027094
		rxfp1/2-L27	SPU_027097
		rxfp1/2-L28	SPU_028324

Table S12. Vertebrate INS/IGF genes (4 species)

	**Medaka (*O. latipes*)**			
**Name**	ins1	ins2	igf1	igf2
**Map location**	scaffold498: 0.025	scaffold223: 0.409	Chr23: 20.44	scaffold1060: 0.013
**Ensembl ID**	ENSORLG00000018432	ENSORLG00000018994	ENSORLG00000016443	ENSORLG00000018930
**Chromosomes***	21	14	23	14?
				missing b-chain, not included in final phylogeny
	**Tetraodon (*T. nigroviridis*)**			
**Name**	ins1	ins2	igf1	igf2
**Map location**	1: 3.4	7: 10.6	19: 1.4	13: 6.6
**Ensembl ID**	ENSTNIG00000004978	ENSTNIG00000004978	ENSTNIG00000012663	ENSTNIG00000010464
	**Human (*H. sapiens*)**			
**Name**	INS	IGF1	IGF2	
**Map location**	Chr11: 2.15	Chr12: 102.79	Chr11: 2.15	
**Ensembl ID**	ENSG00000129965	ENSG00000017427	ENSG00000167244	
	**Chicken (*G. gallus*)**			
**Name**	ins	igf1	igf2	
**Map location**	Chr5: 14.85	Chr1: 57.33	Chr5: 14.88	
**Ensembl ID**	ENSGALG00000006552	ENSGALG00000012755	ENSGALG00000006555	

* putative chromosomes from UTGB database: the medaka genome is not fully assembled
